# Deep Learning Could Diagnose Diabetic Nephropathy with Renal Pathological Immunofluorescent Images

**DOI:** 10.3390/diagnostics10070466

**Published:** 2020-07-09

**Authors:** Shinji Kitamura, Kensaku Takahashi, Yizhen Sang, Kazuhiko Fukushima, Kenji Tsuji, Jun Wada

**Affiliations:** Department of Nephrology, Rheumatology, Endocrinology and Metabolism, Okayama University Graduate School of Medicine, Dentistry and Pharmaceutical Sciences, 2-5-1 Shikata-cho, Okayama-shi, Okayama 700-8558, Japan; me20047@s.okayama-u.ac.jp (K.T.); yizhensang@outlook.com (Y.S.); p7927m3x@s.okayama-u.ac.jp (K.F.); gmd422036@s.okayama-u.ac.jp (K.T.); junwada@okayama-u.ac.jp (J.W.)

**Keywords:** immunofluorescent image, renal pathology, artificial intelligence, deep learning, diabetic nephropathy

## Abstract

Artificial Intelligence (AI) imaging diagnosis is developing, making enormous steps forward in medical fields. Regarding diabetic nephropathy (DN), medical doctors diagnose them with clinical course, clinical laboratory data and renal pathology, mainly evaluate with light microscopy images rather than immunofluorescent images because there are no characteristic findings in immunofluorescent images for DN diagnosis. Here, we examined the possibility of whether AI could diagnose DN from immunofluorescent images. We collected renal immunofluorescent images from 885 renal biopsy patients in our hospital, and we created a dataset that contains six types of immunofluorescent images of IgG, IgA, IgM, C3, C1q and Fibrinogen for each patient. Using the dataset, 39 programs worked without errors (Area under the curve (AUC): 0.93). Five programs diagnosed DN completely with immunofluorescent images (AUC: 1.00). By analyzing with Local interpretable model-agnostic explanations (Lime), the AI focused on the peripheral lesion of DN glomeruli. On the other hand, the nephrologist diagnostic ratio (AUC: 0.75833) was slightly inferior to AI diagnosis. These findings suggest that DN could be diagnosed only by immunofluorescent images by deep learning. AI could diagnose DN and identify classified unknown parts with the immunofluorescent images that nephrologists usually do not use for DN diagnosis.

## 1. Introduction

Lifestyle-related diseases are a big issue for worldwide health. Diabetes mellitus (DM) and chronic kidney diseases are believed to be lifestyle-related diseases. When DM worsens and progresses, diabetic nephropathy (DN) is caused as a microvascular complication. The diagnosis of DN is based on a long history of DM, the presence of other microangiopathies such as diabetic retinopathy and/or diabetic neuropathy (Supplementary Figure S1). Sometimes, differential diagnosis is needed for DM patients if urinary occult blood is clearly observed or a rapid renal function deterioration is demonstrated. In that case, a renal biopsy may be performed to distinguish other glomerular diseases on the view of renal pathology. At the point of renal biopsy evaluation for DN, renal immunofluorescent staining images are considered unimportant because there are few specific features of renal immunofluorescent images in DN. DN is diagnosed typically with nodular lesions, exudative lesions and diffuse lesions in light microscopy images (Supplementary Figure S1). Immunofluorescent images are sensitive in demonstrating the immune complex deposition in the kidney. Immunofluorescence images of DN reveal no specific immune complex deposition because DN is not involved in the immune mechanism pathophysiologically. Therefore, immunofluorescent image analysis for DN has been relatively neglected.

On the other hand, it is expected that artificial intelligence (AI) will be applied to medical diagnosis and treatment [1]. AI is believed to be excellent for image recognition. The application of AI has been studied in various fields such as radiographic imaging [2], cancer invasion [3], drug discovery [4] and ophthalmology [5]. In nephrology, various reports are expected, such as the prediction of acute kidney injury from a patient’s big data [6], segmentation of renal tissue images [7] and renal function prediction from renal echo images [8]. However, AI performs the diagnosis or treatment by using the data that human doctors use. There are few reports on whether AI could diagnose or predict the diseases using the data that human doctors do not employ.

Here, we examined whether DN can be diagnosed by AI based on immunofluorescent images that are rarely used in clinical practice by human doctors. AI excels in the pattern recognition of images and data, and may be able to extract and discriminate features that are barely discernible by human eyes.

## 2. Materials and Methods

### 2.1. Patients Information

This study was approved by the committee of Okayama University at August 2^nd^, 2019 (approved #: ken1908-008). This study is a retrospective observational study. As for informed consent, the contents of the research are posted on our department homepage and in the hospital, and public informed consent was provided. Kidney samples consisted of needle biopsy samples from patients that were hospitalized to undergo the examination of renal biopsy in Okayama University hospital from January 2008 to May 2018. The number of renal biopsy patients is 885 from 2012–2018 in the Okayama University hospital. During the 2012–2018 period, the number of DN renal biopsy patients was 31 in the Okayama University hospital. For comparison, 130 non-DN patients’ images were collected in 2018. Among them, we excluded 78 patients because of renal transplantation episode biopsy, complicated multiple nephropathy and uncertain diagnosis. The diagnosis of remaining 52 non-DN patients was shown in Supplementary Figure S2. Almost a quarter of them are IgA nephropathy patients (Supplementary Table S1). The diagnosis of the renal disease was decided by the nephrologists with the discussion in conference by using the patient’s medical history, physical information, clinical examination information and pathological findings including immunofluorescent images, light microscopy images and electron microscopy images. 

### 2.2. Renal Immunofluorescent Images

The kidney samples were obtained by renal biopsy from the patients administrated in Okayama University Hospital. We made frozen sections from renal biopsy samples, and cut at 4 um in a cryostat. We stained the frozen sections by fluorescein isothiocyanate (FITC)-conjugated antibodies in a moist chamber for 1 h. FITC-conjugated goat anti-human IgA, IgM, C3, C1q and fibrinogen were purchased from MP biomedicals, LLC, and FITC-conjugated goat anti-human IgG was purchased from Medical and Biological Laboratories Co., LTD. The images were obtained by fluorescence microscopes (Olympus, Japan). We collected only glomerular immunofluorescent images from each section. To analyze each glomerular image by deep learning, we aligned each size of glomerular images for comparison.

### 2.3. Data Preprocessing

The image data were obtained as JPEGs or Tiff files. We changed the resolution of the files from 2776 × 2074 pixel to 256 × 256 pixels. After the conversion, we converted the JPEG file into a PNG file for analysis. 

### 2.4. Deep Learning

Python was used as the programming language, and the environment was Microsoft’s visual studio code and neural network console (Sony Network Communications Inc., Tokyo, Japan.). The input was six types of renal immunofluorescent images of IgG, IgA, IgM, C3, C1q, and fibrinogen converted as previously described. The renal pathological images are classified into training images and test images at a ratio of 8:2. The test images are shown in Supplementary Figure S3. We performed supervised training for deep learning.

### 2.5. Statistical Analysis 

Statistical analysis was performed by JMP (SAS Institute Inc. version 11.0.0 for Windows software, Tokyo, Japan). Statistical significance was defined by one-way analysis of variance (ANOVA) with Student’s *T*-test. Data are shown as the mean ± SE. Significance was defined as *p* < 0.05. In addition, to determine the cut-off value of the DN diagnosis, a receiver operating characteristic (ROC) curve was constructed using statistical analysis software JMP.

## 3. Results

### 3.1. Overview of Computer Schema of Deep Learning

**Figure 1:**
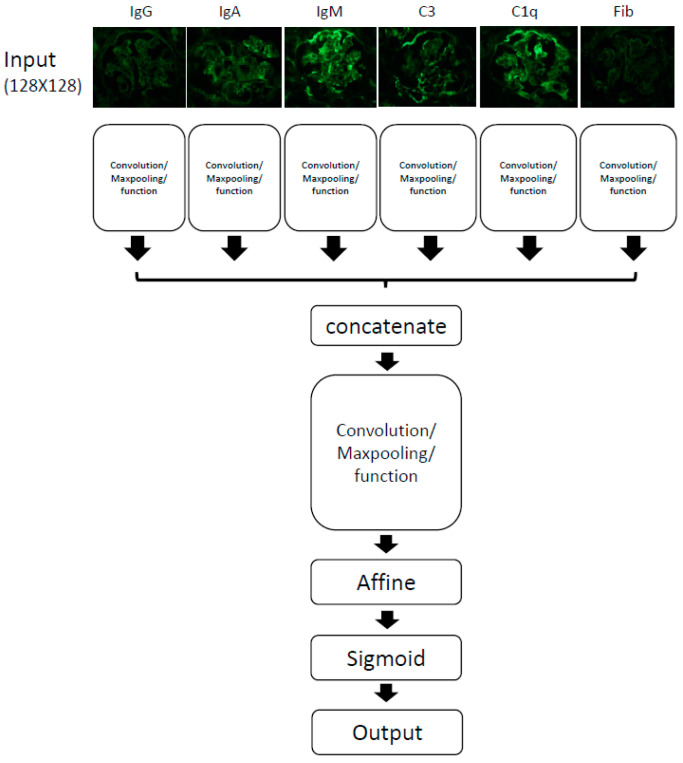
The overview of convolution neural networkprogram. We used input data as six types of renal immunofluorescent images, IgG, IgA, IgM, C3, C1q and Fibrinogen (Fib).

An overview of the computational schema is shown (Figure 1). We input six kinds of immunofluorescent images, IgG, IgA, IgM, C3, C1q and Fibrinogen. Each image resolution is 256 × 256 pixel. Each image was analyzed, and six types of data were integrated, analyzed again and connected to the output. We used the software Neural Network Console provided by Sony Inc. This software automatically adds or deletes some layers to adjust parameters, obtaining an optimum result. Using this software, we created 419 different types of programs in this study. We evaluated them with a learning curve for each program. Some programs did not work well, and we harvested some better programs for this study.

### 3.2. AI Could Diagnose DN from Immunofluorescent Images

**Figure 2:**
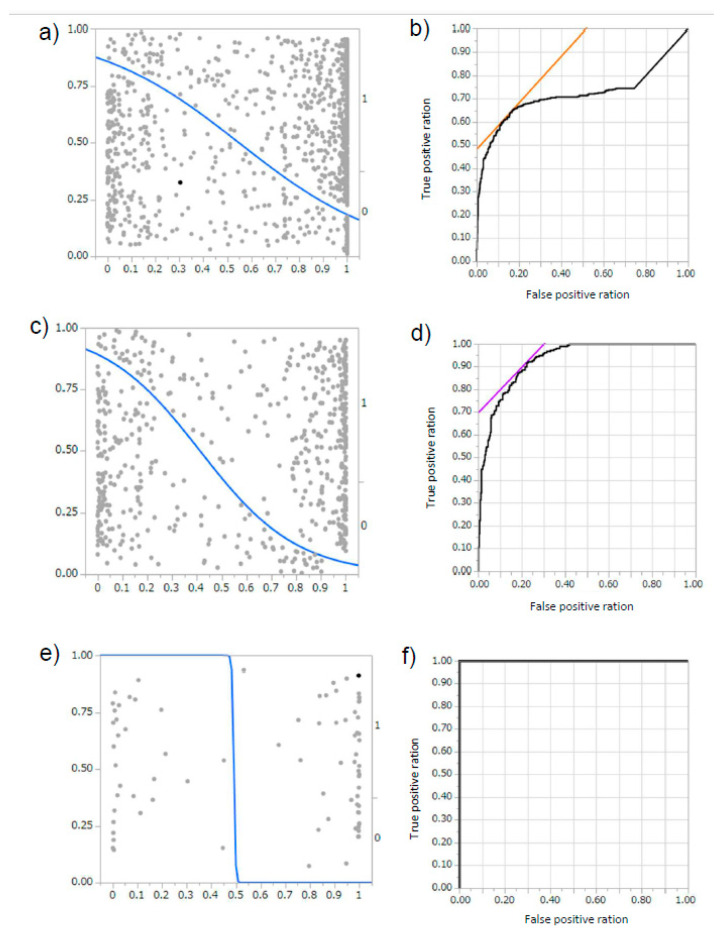
The area under the curve (AUC) for each program. (**a**,**b**) For 419 programs, (**a**) Logistic finding curve; diabetic nephropathy (DN): 0, non-DN: 1, (**b**) receiver operating characteristic (ROC) curve; AUC 0.71807, *p* < 0.0001, R^2^ 0.2213. (**c**,**d**) For 39 programs with accuracy above 60% average, (**c**) Logistic finding curve; DN: 0, non-DN: 1, (**d**) ROC curve; AUC 0.92914, *p* < 0.0001, R^2^ 0.4586. (**e**,**f**) For six complete diagnosis program, (**e**) Logistic finding curve; DN: 0, non-DN: 1, (**f**) ROC curve; AUC 1.000, *p* < 0.0001, R^2^ 1.000.

A total of 419 programs were trained using the immunofluorescent images obtained in our hospital (representative: Supplementary Figure S4, a: schema of program, b: learning curve, c: result of diagnosis). Their programs ranged in accuracy from 30% to as high as 100%. The total area under the curve (AUC) of the diagnostic rate of all the created programs was 0.71807, R^2^ 0.2213, *p* < 0.0001 (Figure 2a,b). In addition, among the obtained programs, we analyzed the 39 programs where the accuracy ratio was 60% or more. In these extracted programs, the accuracy rate was 83.28 ± 11.64%, the precision rate was 80.56 ± 21.83%, and the recall rate was 79.87 ± 15.65%, and the AUC was 0.92914, R^2^ 0.4586, *p* < 0.0001 (Figure 2c,d). Six programs showed 100% accuracy, precision, and recall, and the AUC was 1.000, R^2^ 1.000, *p* < 0.0001 (Figure 2e,f). This indicates that AI could automatically extract features from limited image information, and that the judgment is reproduced at high rates even in test data.

### 3.3. The Differences of the Diagnosis among DN Immunofluorescent Images

**Figure 3:**
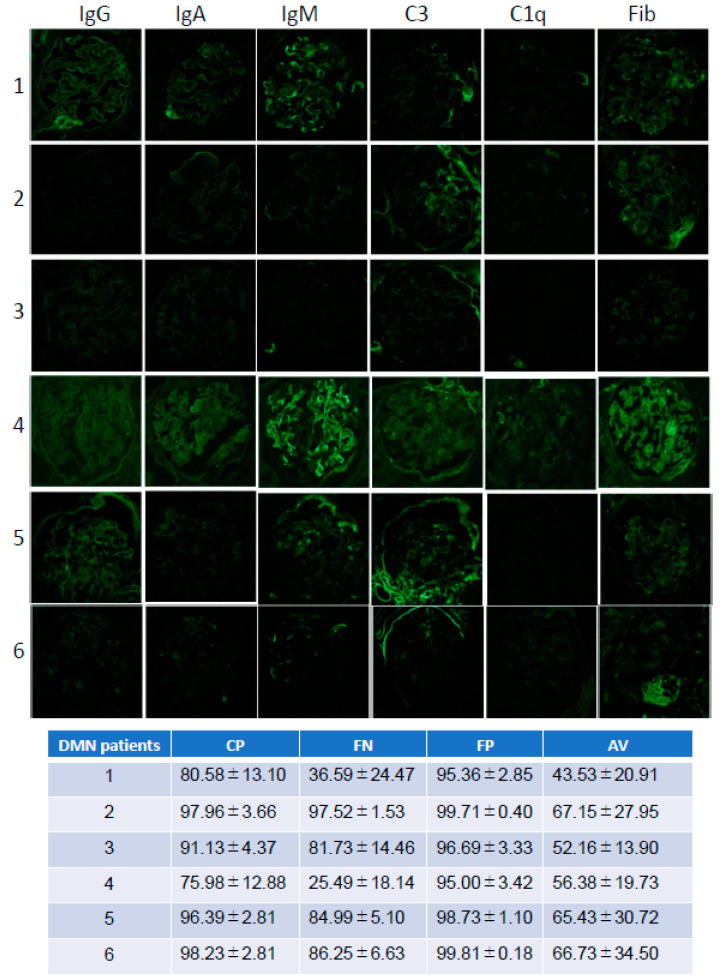
Test DN images and each program diagnosis. Patient number; 1, 2, 3, 4, 5, 6 Immunofluorescent imaging type; IgG, IgA, IgM, C3, C1q, Fibrinogen (Fib). Each test images diagnosis (%) for each program (CP: completely diagnosis program, FN: False negative program, FP: False positive program, AV: average accuracy program).

Next, the DN images used in the test dataset were analyzed in the point of accuracy. We used test image data, which consisted of, representatively, six DN patients’ images (Figure 3). We compared the accuracy with four types programs, the complete diagnosis program (CP: Supplementary Figures S5 and S6a,b), false negative program (FN: Supplementary Figures S7 and S8a,b), false positive program (FP: Supplementary Figures S9 and S10a,b), and average accuracy program (AV: Supplementary Figures S11 and S12a,b). Regarding each accuracy, patient #1 and patient #4 showed a lower accuracy ratio compared to other DN patients. On the FN program, patient #1 and #4 were not diagnosed as DN. On the other hand, in terms of picking up DN, the FP program could pick up all DN images. However, the FP program diagnosed interstitial nephritis and antineutrophil cytoplasmic antibody (ANCA)-related nephritis as DN (Supplementary Figure S10b). In the AV program, patient #1 did not diagnose as DN, and other DN patients diagnosed as DN slightly above the diagnosis line. These results suggest that AI could diagnose DN the same as a human could. 

### 3.4. Lime Analysis

**Figure 4:**
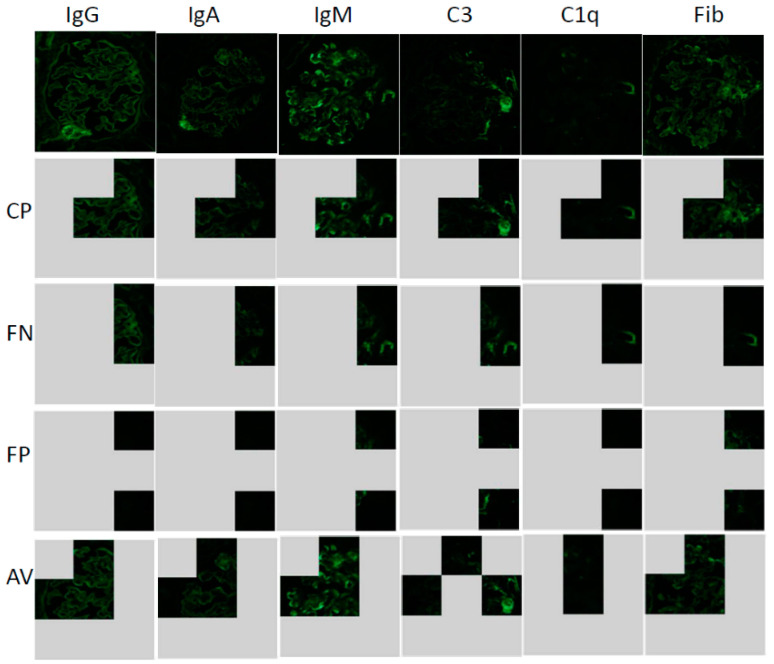
Local interpretable model-agnostic explanations (Lime) analysis for each program for patient #1 image. Lime analysis: AI extracted focused area for each program: Immunofluorescent imaging type; IgG, IgA, IgM, C3, C1q, Fibrinogen (Fib); Each program (CP: completely diagnosis program, FN: False negative program, FP: False positive program, AV: average accuracy program).

Next, to determine the characteristic findings in glomerular changes on DN, we examined which parts of image areas were the main focus under AI diagnosis by using the local interpretable model-agnostic explanations (Lime) analysis. The Lime analysis could show the part where AI mainly makes a decision. We chose representative images of patient #1 (Figure 3). The CP program focused on a part of the central site and the periphery (Figure 4 CP, Supplementary Figure S13), In addition, another CP program focused on the periphery (Supplementary Figure S14). These results suggest that characteristic findings may be located at the periphery for glomeruli of DN. In programs that produce false negatives (FN), judgments were often made only at the margins (Figure 4 FN). Conversely, even in programs that produce false positives (FP), judgments were based only on the margins (Figure 4 FP). In programs with both false positives and false negatives (AV), judgements were made at the center and margins (Figure 4 AV). These results suggest that, as a characteristic finding, judgment was made mainly on the change of the snare, and the judgment was compared with the central part.

### 3.5. The Diagnostic Comparison between Nephrologist and AI

**Figure 5:**
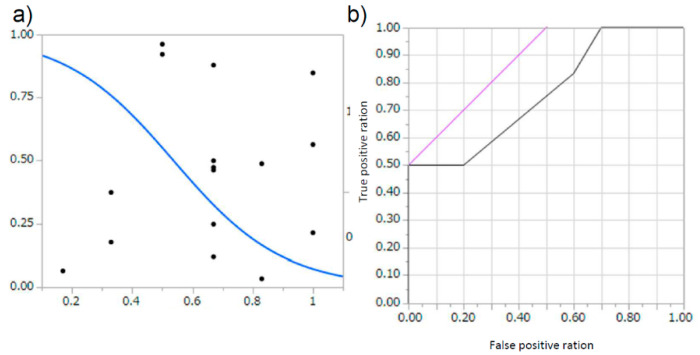
The differences of the diagnosis compared between AI and nephrologist. (**a**,**b**) For evaluation of six nephrologists, (**a**) Logistic finding curve; DN: 0, non-DN: 1, (b) ROC curve; AUC 0.75833, *p* = 0.0326, R^2^ 0.2156.

To compare the differences between humans and AI, we provided the diagnosis test to nephrologists, using the same test data. Among 39 previously described programs that showed the accuracy above 60%, the diagnosis rate of the test images by the AI was 83.28 ± 11.64% for the accuracy ratio, 80.56 ± 21.83% for the precision ratio and 79.87 ± 15.65% for the recall ratio (Figure 2c,d). On the other hand, the diagnosis rate of the test images by the nephrologist was 67.50 ± 6.12% for the correct answer rate, 62.62 ± 3.85% for the precision rate, and 67.26 ± 9.96% for the recall rate (Figure 5a,b). These results suggest that AI could diagnose the DN in a superior way to nephrologists. 

## 4. Discussion

The purpose of this study is to examine whether AI can automatically extract features from images that doctors do not consider important in diagnosis by deep learning, and whether AI could make a diagnosis.

DN is usually diagnosed with reference to the long-term history of diabetes and the presence of microangiopathic complications such as diabetic retinopathy. When it is necessary to differentiate from other renal diseases in DM patients, because of the preference of atypical features in DN such as hematuria, sudden onset of proteinuria, and a change in renal function, a renal biopsy is performed and the patients are diagnosed from renal pathology. In DN, characteristic lesions such as diffuse lesions, exudative lesions, and nodular lesions are observed in light microscopy, however, the immunofluorescent images are not used because there are no significant parts. AI can automatically extract and classify features from the immunofluorescent images. From the Lime analysis, AI pointed out the changes in the peripheral vessel loop. At the peripheral vessels loop, we could observe the changes of vessel loop thickening and/or wrinkling in the light microscopy. However, the changes were very subtle in immunofluorescent images, thus we could not observe any significance compared to other glomeruli with nephritis using human eyes.

However, in the analysis of AI, there are still many unclear points about the judgment during the process, and this is said to be the so-called AI black box problem. In the future, we hope that a method will be created that allows humans to understand AI decisions. In this study, we did not examine the difference in the diagnosis rate when using light microscopy images. AI may develop to be better for diagnosis with light microscopic images because there are significant diagnostic points in light microscopic images. 

As a study limitation, AI diagnosis is performed only from immunofluorescent images that were small datasets from limited patient images. In order to validate them, verification will be necessary by adding data from other hospitals. In addition, human doctors diagnose comprehensively with clinical course, clinical findings and clinical images, such as light microscope images and electron microscope images. To compare the differences between human and AI, further examination is needed.

This study showed that AI extracts characteristic findings even from data that people do not normally use. This indicates that when a medical doctor makes a clinical diagnosis, AI can independently extract the findings that human doctors find difficult to notice. This study suggested that AI is not a threat to clinicians, but a partner that points out the difficult points which humans do not notice. 

## Supplementary Materials

The following are available online at https://www.mdpi.com/2075-4418/10/7/466/s1. Supplemental Figure S1; The diagnosis method for diabetes mellites nephropathy (DN). Supplementary Figure S2; Enrolment. All patients underwent renal biopsy in Okayama University Hospital. Supplementary Table S1; compared other nephropathy and patient number. Supplementary Figure S3a-c; (a) Test data set for DN. (b) Test data set for non DN. A: C3 nephropathy, B: FGS (focal segmental glomerular sclerosis), C: MPGN (membranoproliferative glomerulonephritis), D: TBM (thin basement membrane disease) E: MCNS (micro change nephrotic syndrome), F: interstitial nephritis, G: ANCA (antineutrophil cytoplasmic antibody) related disease. (c) Test data set for non DN. H: MN (Membranous nephropathy), I: IgA-N (IgA nephritis), J: IgA-N. Supplementary Figure S4; Representative CNN data. (a) schema of CNN; (b) learning curve; (c)evaluation data (y = 0: DN, y = 1: non DN, y’ = 0: estimated DN, y’ = 1: estimated non DN). Supplementary Figure S5; Representative completely diagnosis (CP) CNN data. (a) schema of CNN; (b) learning curve; (c) evaluation data (y = 0: DN, y = 1: non DN, y’ = 0: estimated DN, y’ = 1: estimated non DN). Supplementary Figure S6a, b; (a) representative completely (CP) DN diagnosis ratio for DN patients’ images. (b) representative completely (CP) DN diagnosis ratio for non DN patients’ images. Supplementary Figure S7; Representative false negative diagnosis (FN) CNN data. (a) schema of CNN; (b) learning curve; (c) evaluation data (y = 0: DN, y = 1: non DN, y’ = 0: estimated DN, y’ = 1: estimated non DN). Supplementary Figure S8a, b; (a) representative false negative (FN) DN diagnosis ratio for DN patients’ images. (b) representative false negative (FN) DN diagnosis ratio for non DN patients’ images. Supplementary Figure S9; Representative false positive diagnosis (FP) CNN data. (a) schema of CNN; (b) learning curve; (c) evaluation data (y = 0: DN, y = 1: non DN, y’ = 0: estimated DN, y’ = 1: estimated non DN). Supplementary Figure S10a, b; (a) representative false negative (FN) DN diagnosis ratio for DN patients’ images. (b) representative false negative (FN) DN diagnosis ratio for non DN patients’ images. Supplementary Figure S11; representative average diagnosis (AV) CNN data; (a) schema of CNN; (c) learning curve; (c) evaluation data (y = 0: DN, y = 1: non DN, y’ = 0: estimated DN, y’ = 1: estimated non DN). Supplementary Figure S12a, b; (a) representative average (AV) DN diagnosis ratio for DN patients’ images. (b) representative average (AV) DN diagnosis ratio for non DN patients’ images. Supplementary Figure S13; Lime analysis for patient #1 images with completely diagnosis program #1. Supplementary Figure S14; Lime analysis for patient #1 images with another completely diagnosis program #6.

## Abbreviations

DN	Diabetic nephropathy
AI	Artificial intelligences
AUC	Area under the curve
Lime	Local interpretable model-agnostic explanations
CNN	Convolution neural network
ANCA	Antineutrophil cytoplasmic antibody
Fib	Fibrinogen
