# Supplementary material for: Deep Learning Could Diagnose Diabetic Nephropathy with Renal Pathological Immunofluorescent Images
Source: Diagnostics (Basel). 2020 Jul 9;10(7):466. doi: 10.3390/diagnostics10070466 (PMC7400564; doi:10.3390/diagnostics10070466)
Supplement: Supplementary file 1 [file diagnostics-10-00466-s001.pdf]

Supplemental Fig.S1

Diagnosis of DN

・long term diabetes mellites (more than 5 years)
・Presence of complications, such as DM retinopathy or DM neuropathy
・Presence of persistent proteinuria, such as microalbuminuria. In addition, other glomerular diseases are excluded
・No noticeable hematuria
・ Initially increased glomerular filtration rate and / or kidney enlargement

➕

Renal pathology

Immunofluorescent image analysis

Light microscopic image analysis
Characteristic findings
・Nodular lesion
・Exudative lesion
・Diffuse lesion

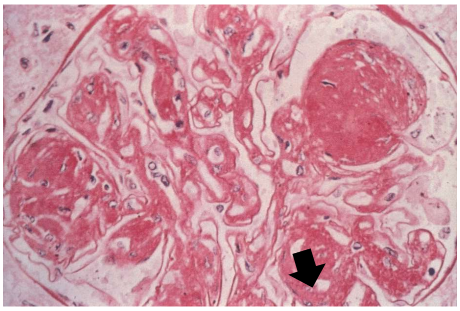

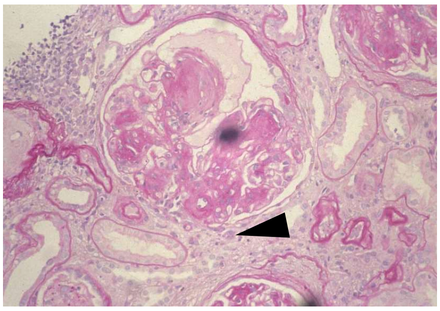

Arrow: Nodular lesion, Arrow head: Exudative lesion

Supplemental Fig.S1
The diagnosis method for diabetes mellites nephropathy (DN)

Supplemental Fig.S2

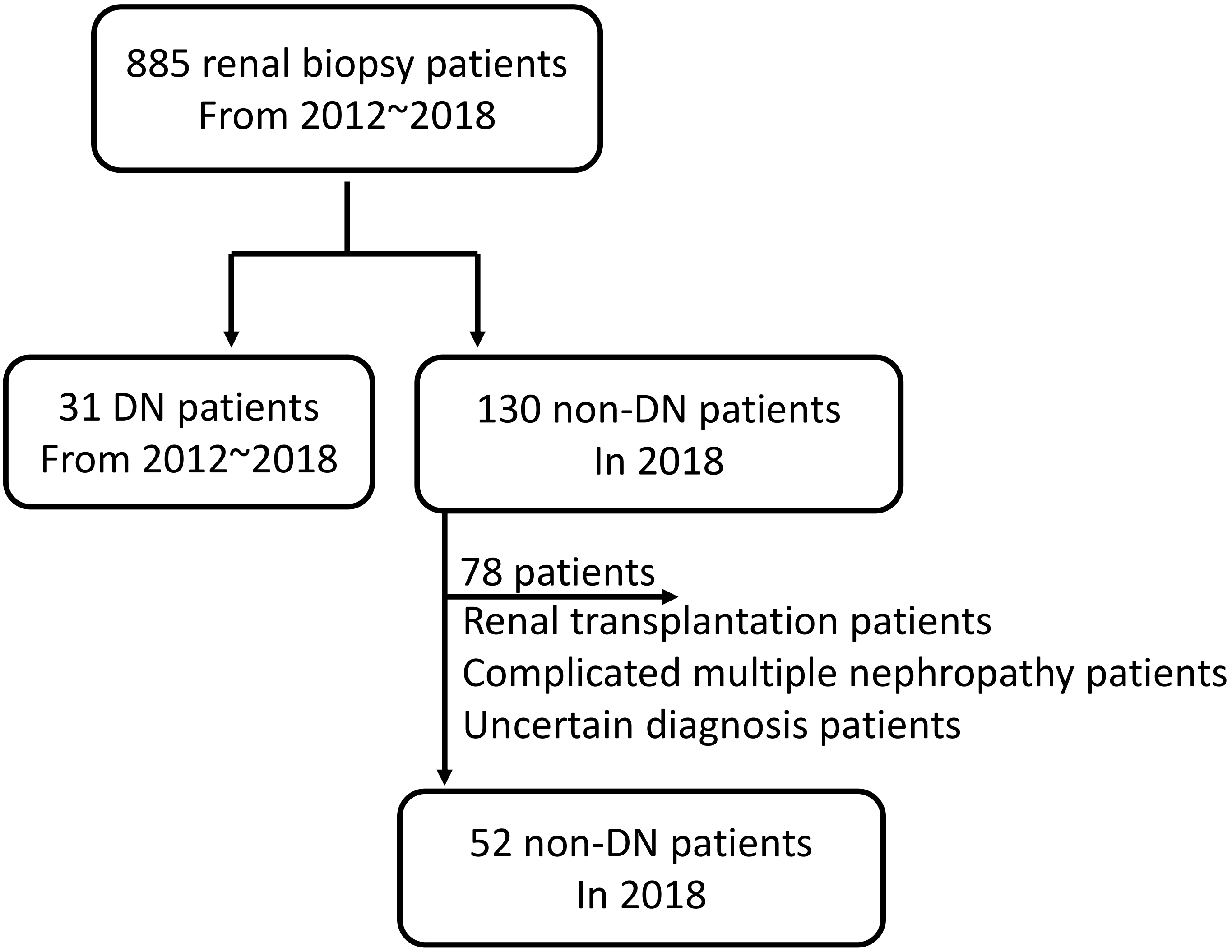

supplementary Fig. S2
Enrolment
All patients underwent renal biopsy in Okayama University Hospital.

Supplemental Table.ST1

Diagnosis	Patients number
IgA nephropathy	17
Membranous nephropathy	9
ANCA related nephropathy	5
Membranous proliferative glomerular nephropathy	4
Minor change nephrotic syndrome	3
Tubulointerstitial nephropathy	3
Thin basement membrane disease	2
Immunotactoid disease	1
Focal segmental glomerular sclerosis	1
Acute tubular necrosis	1
Glomerular sclerosis	1
C3 nephropathy	1
Amyloid disease	1
Obesity related nephropathy	1
Total	52

supplementary Table. ST1
compared other nephropathy and patient number

Supplemental Fig. S3a

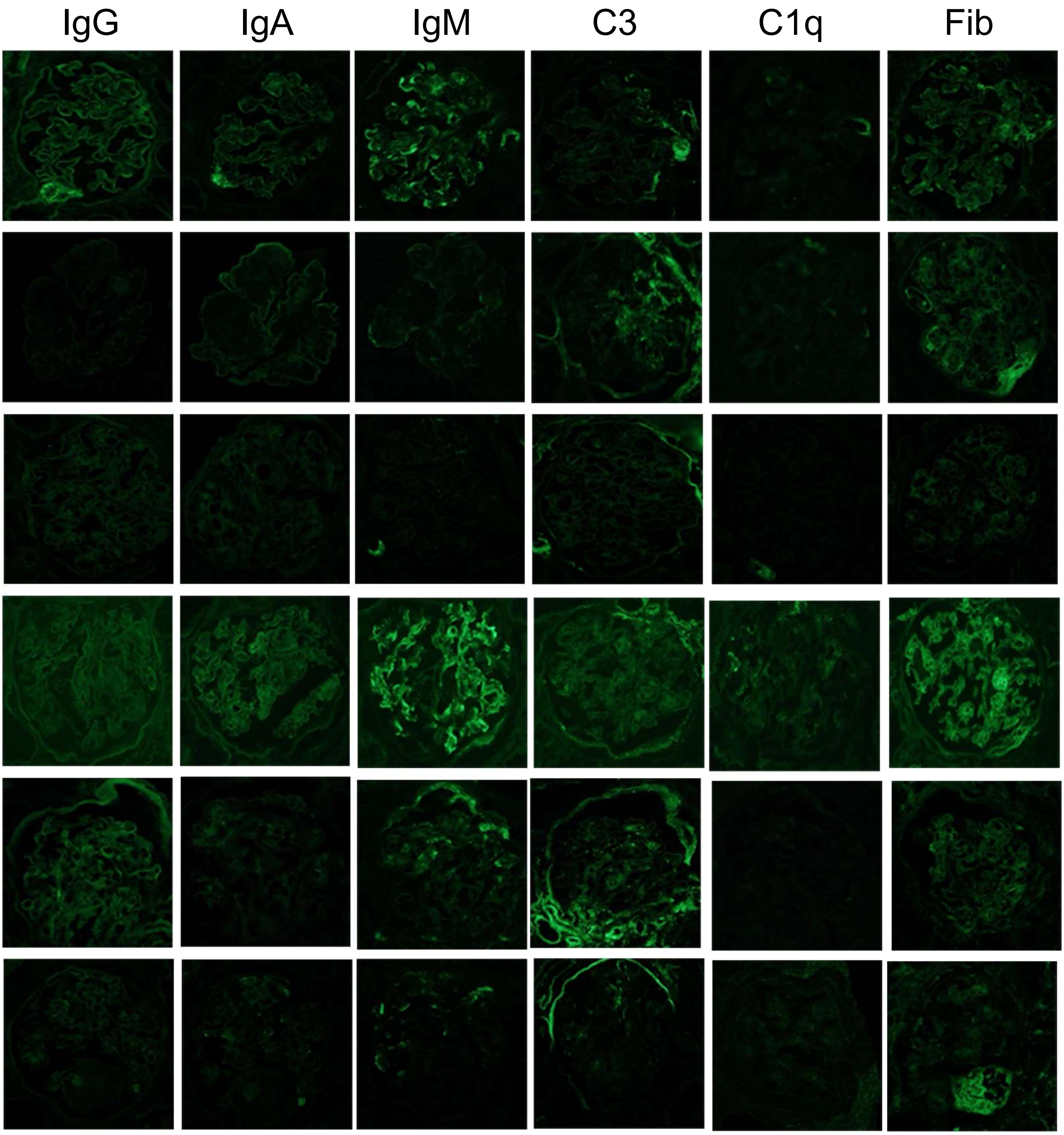

supplementary Figure. S3a
Test data set for DN

Supplemental Fig.S3b

IgG IgA IgM C3 C1q Fib

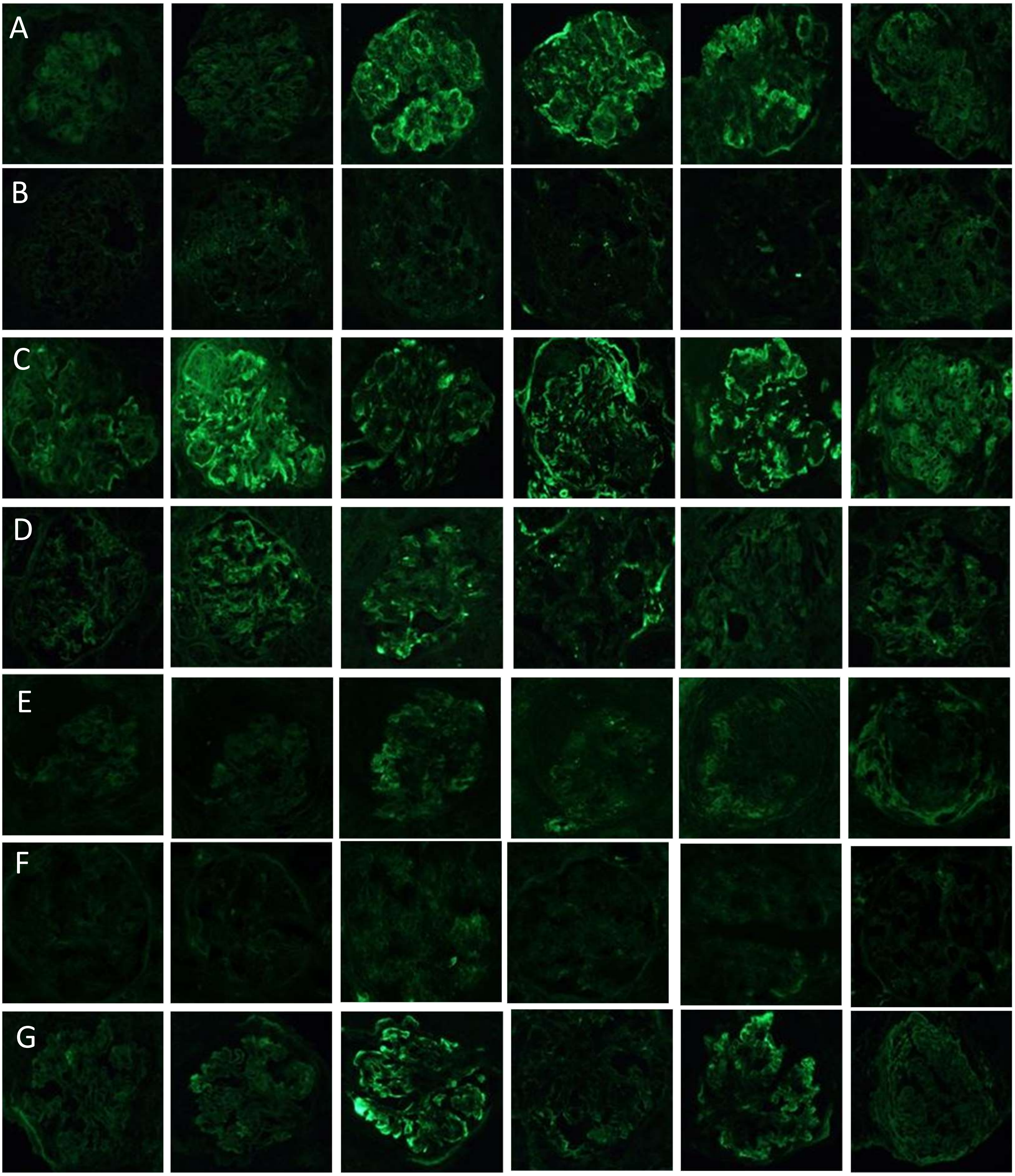

supplementary Figure. S3b

Test data set for non DN: A: C3 nephropathy, B: FGS (focal segmental glomerular sclerosis), C: MPGN (membranoproliferative glomerulonephritis), D: TBM (thin basement membrane disease) E: MCNS (micro change nephrotic syndrome), F: interstitial nephritis, G: ANCA (antineutrophil cytoplasmic antibody) related disease

Supplemental Fig.S3c

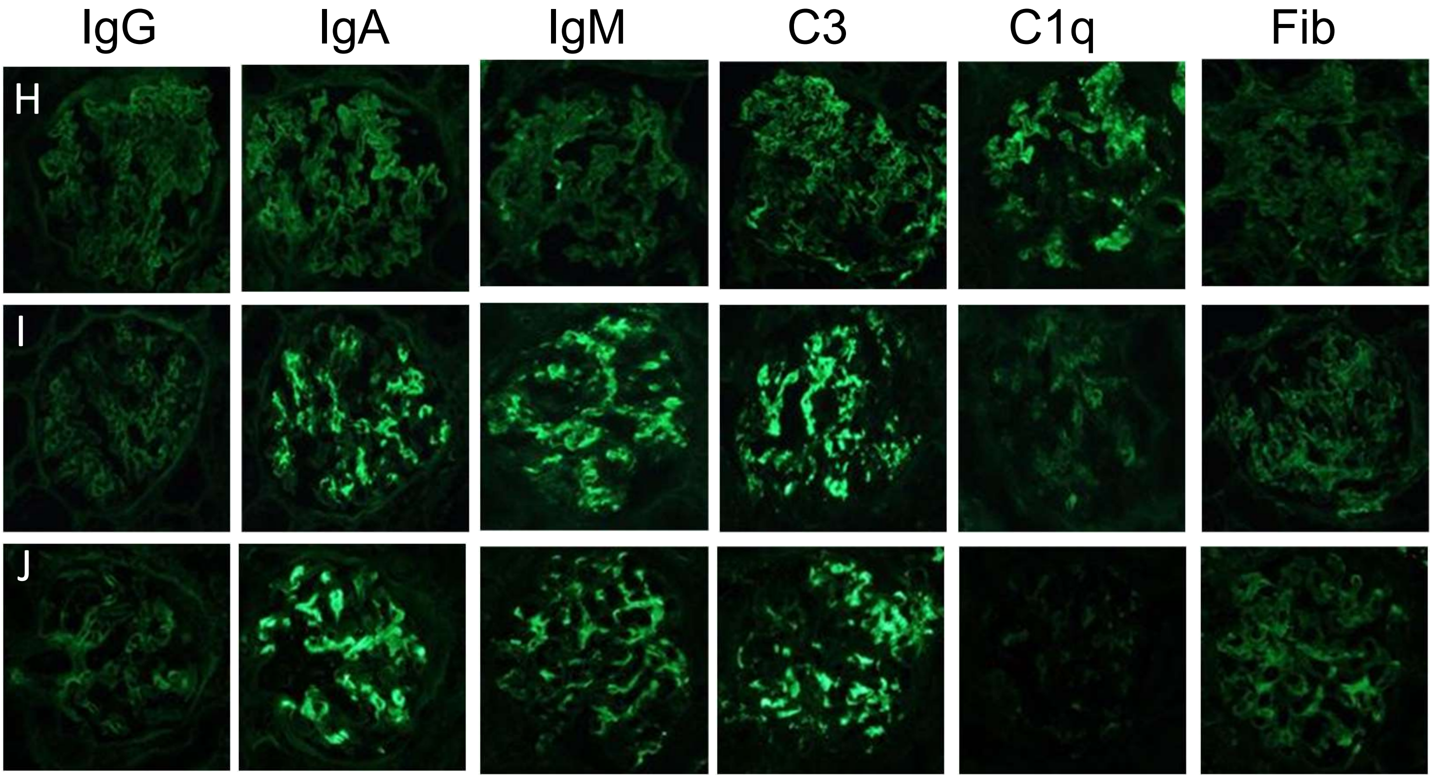

H: MN, I: IgA-N, J: IgA-N

supplementary Figure. S3c
Test data set for non DN
H: MN (Membranous nephropathy), I: IgA-N (IgA nephritis), J: IgA-N

Supplemental Fig.S4

a)

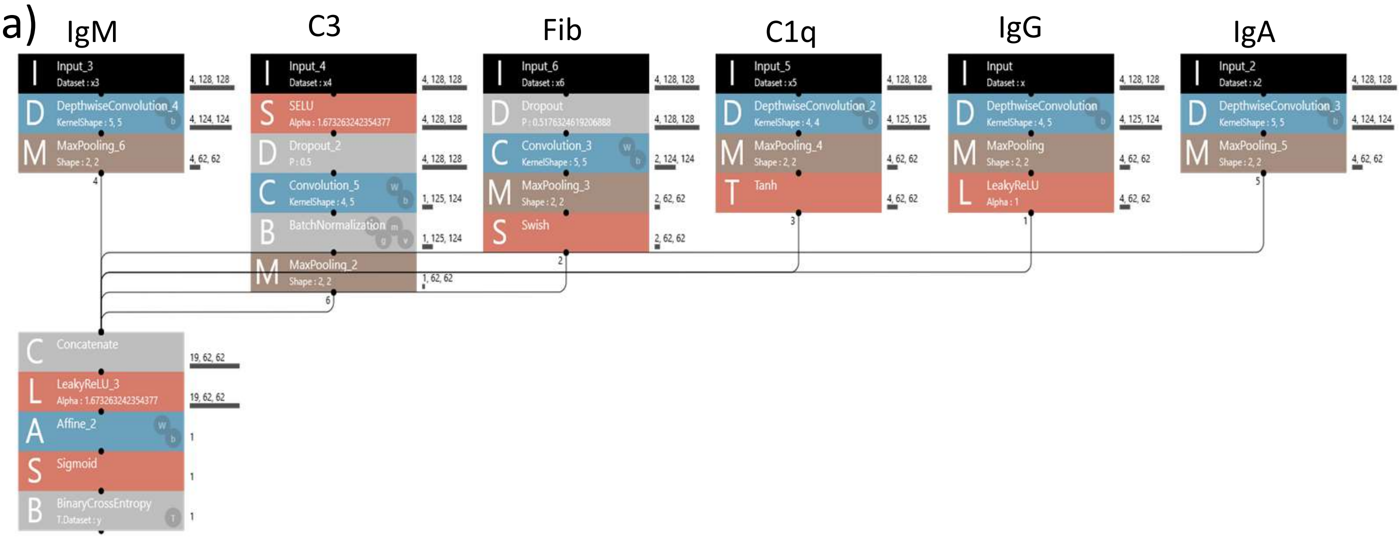

b)

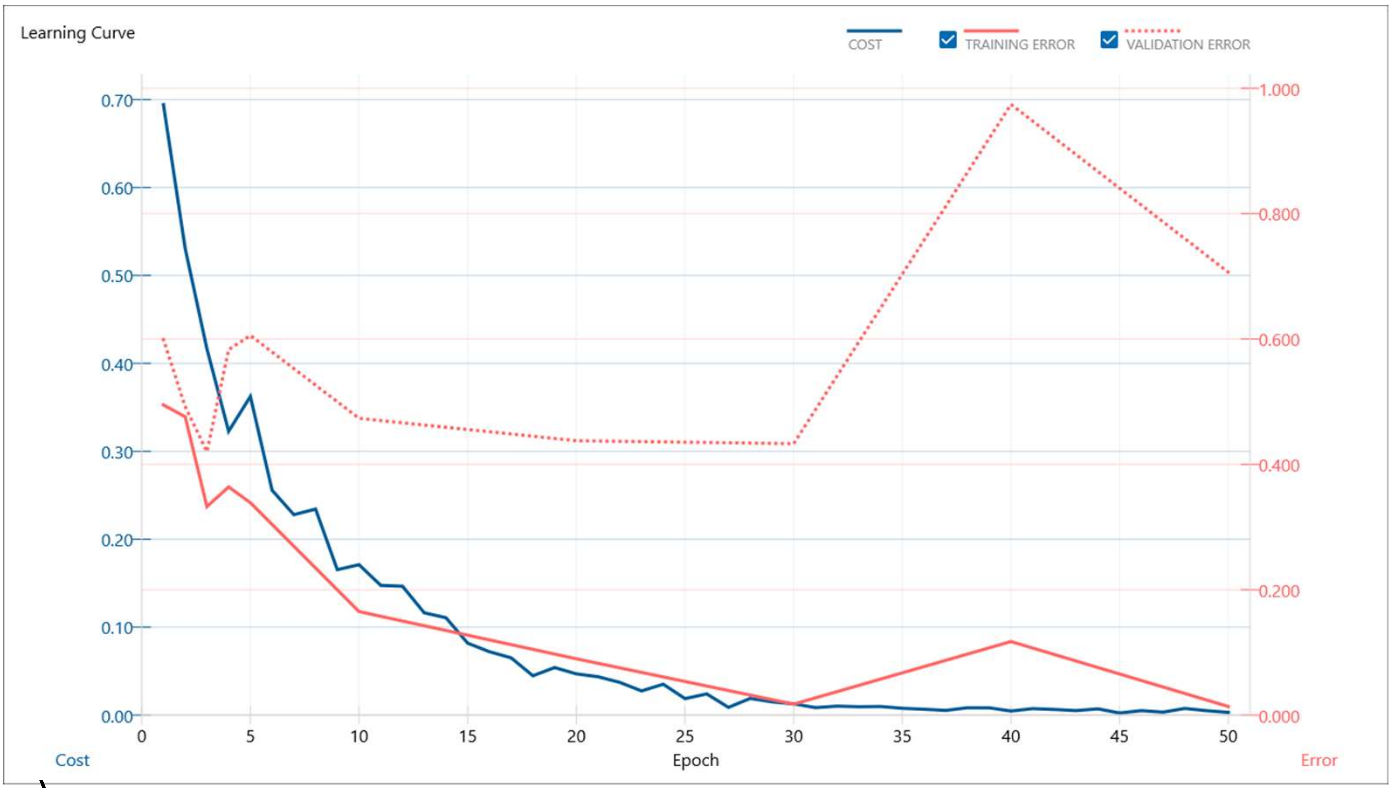

c)

	y'=0	y'=1	Recall
y=0	4	2	0.6666
y=1	2	8	0.8
Precision	0.6666	0.8	
F-Measures	0.6666	0.8	

Accuracy	0.75
Avg.Precision	0.7333
Avg.Recall	0.7333
Avg.F-Measures	0.7333

supplementary Figure. S4

representative CNN data

a) schema of CNN, b) learning curve, c) evaluation data

b) (y=0: DN, y=1: non DN, y`=0: estimated DN, y`=1: estimated non DN)

Supplemental Fig.S5

CP

a)

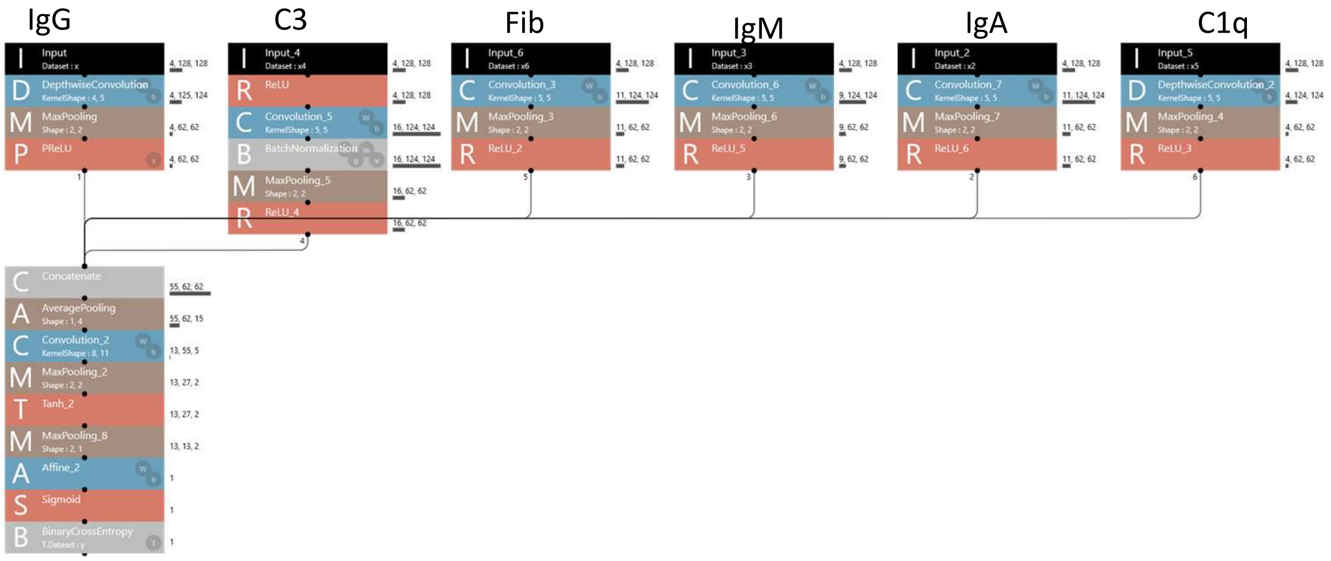

b)

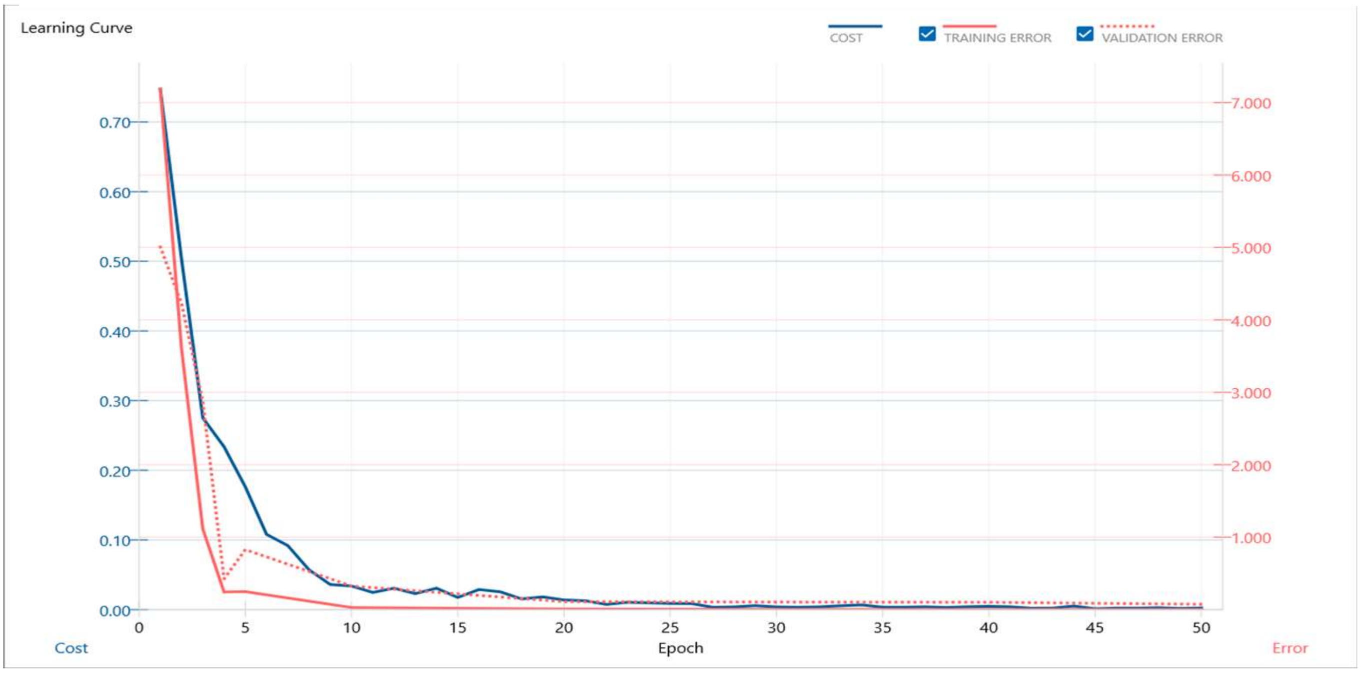

c)

	y'=0	y'=1	Recall
y:label=0	6	0	1
y:label=1	0	10	1
Precision	1	1	
F-Measures	1	1	

Accuracy	1
Avg.Precision	1
Avg.Recall	1
Avg.F-Measures	1

supplementary Figure. S5

representative completely diagnosis (CP) CNN data

a) schema of CNN, b) learning curve, c) evaluation data

(y=0: DN, y=1: non DN, y`=0: estimated DN, y`=1: estimated non DN)

Supplemental Fig.S6a

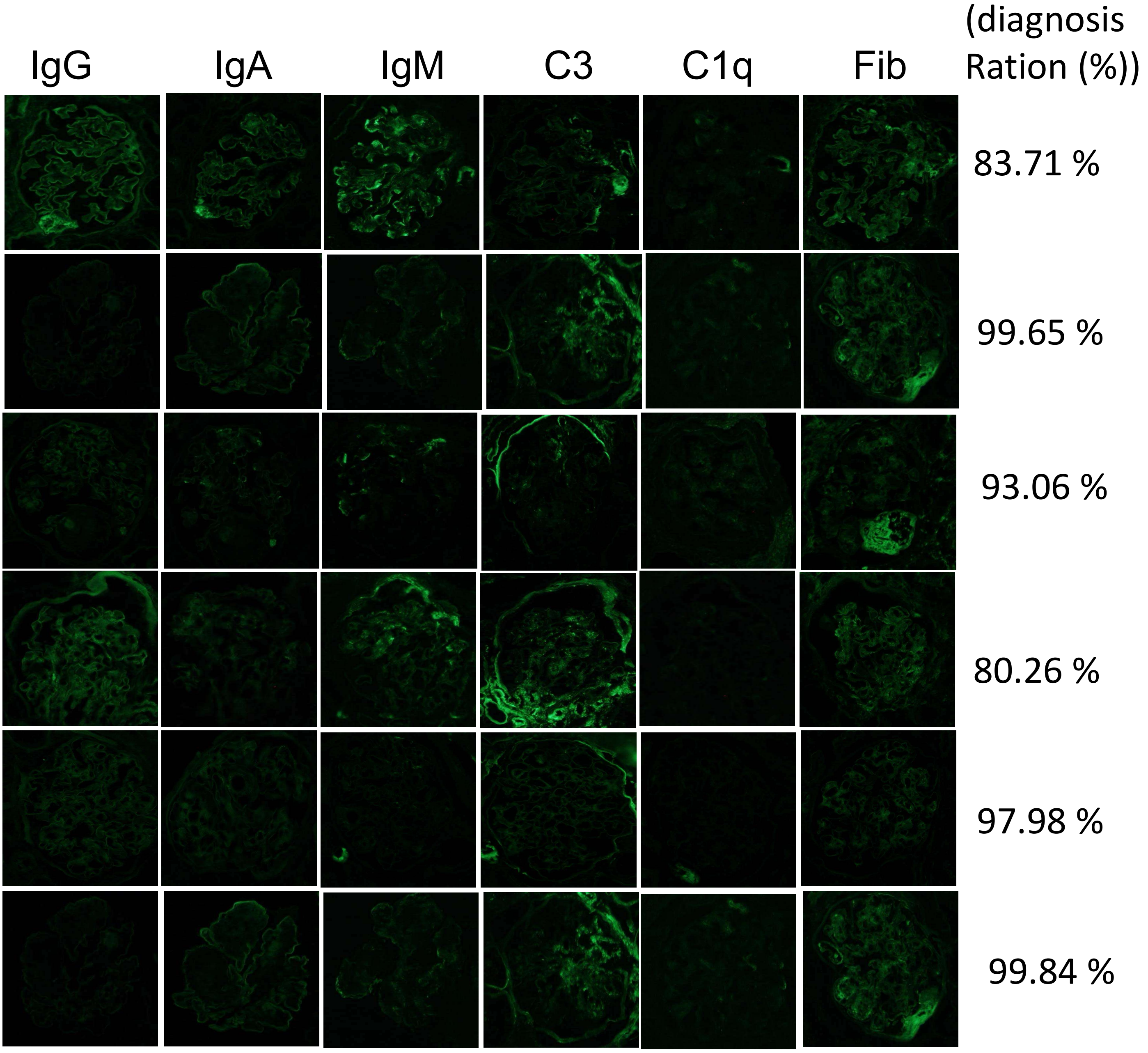

supplementary Figure. S6a
representative completely (CP) DN diagnosis ration for DN patients' images

Supplemental Fig.S6b

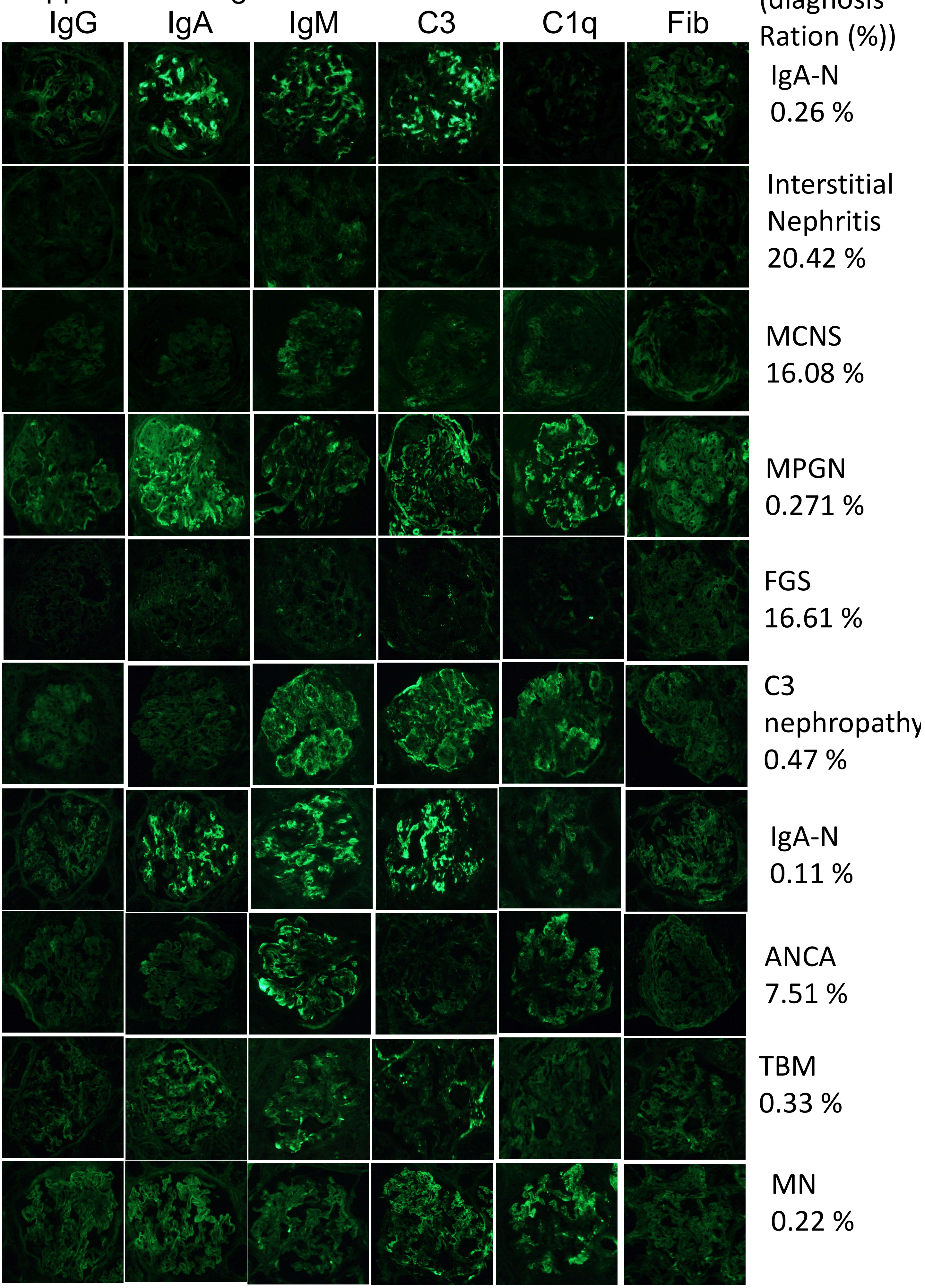

supplementary Figure. S6b
representative completely (CP) DN diagnosis ration for non DN patients' images

Supplemental Fig.S7 FN

a)

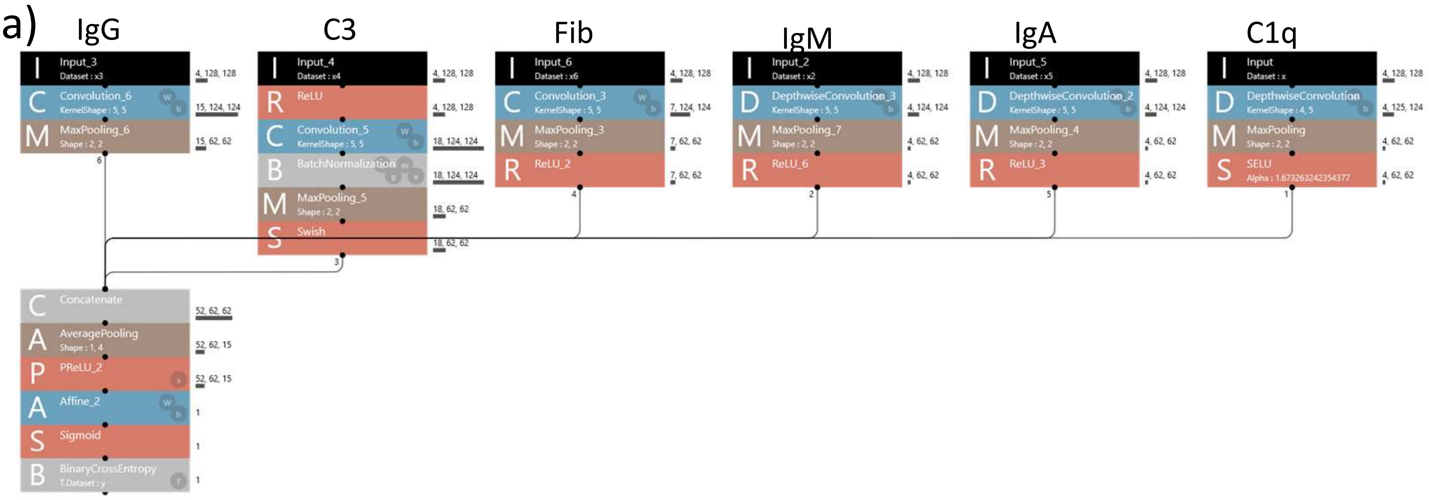

b)

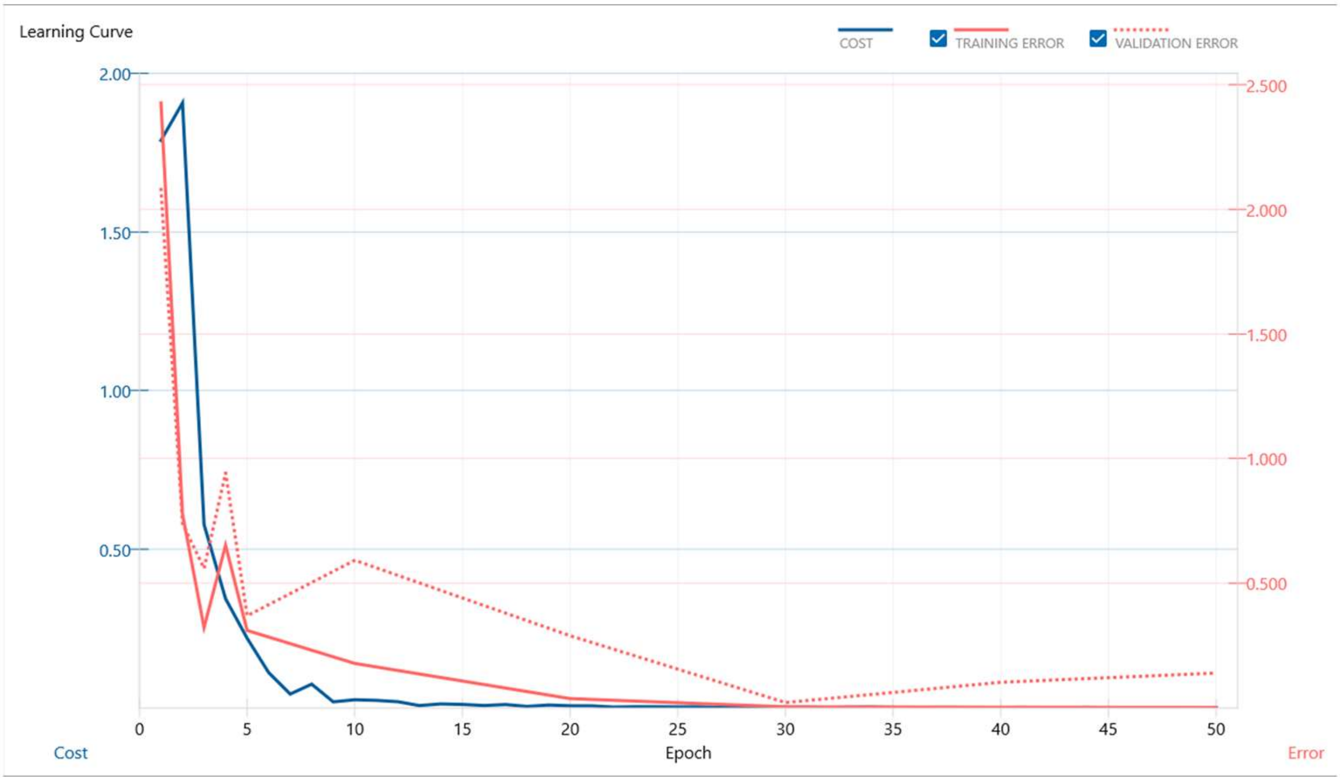

c)

	y'=0	y'=1	Recall
y=0	4	2	0.6666
y=1	0	10	1
Precision	1	0.8333	
F-Measures	0.7999	0.909	

Accuracy	0.875
Avg.Precision	0.9166
Avg.Recall	0.8333
Avg.F-Measures	0.8545

supplementary Figure. S7
representative false negative diagnosis (FN) CNN data
a) schema of CNN, b) learning curve, c) evaluation data
(y=0: DN, y=1: non DN, y`=0: estimated DN, y`=1: estimated non DN)

Supplemental Fig.S8a

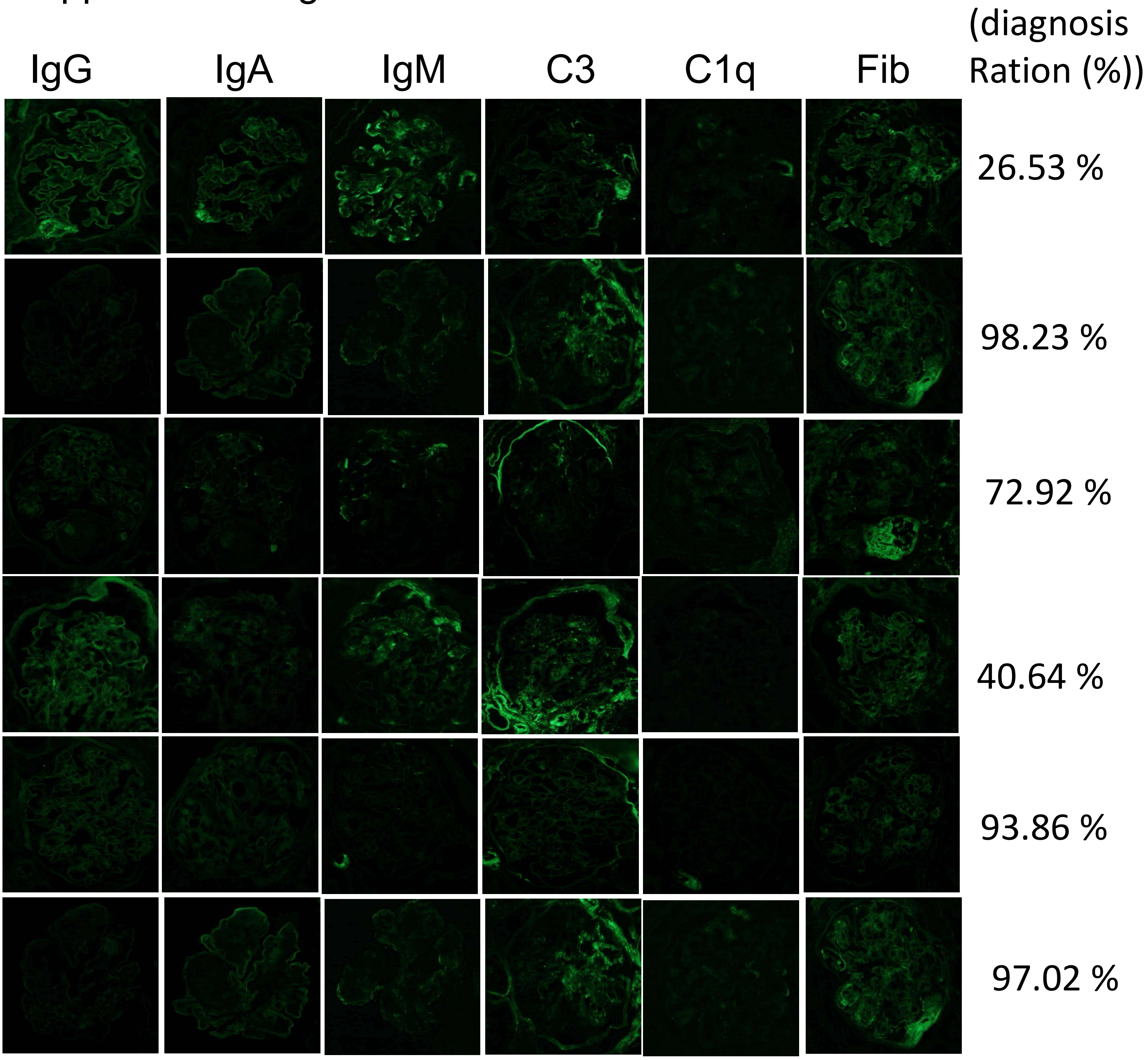

supplementary Figure. S8a
representative false negative (FN) DN diagnosis ration for DN patients' images

Supplemental Fig.S8b

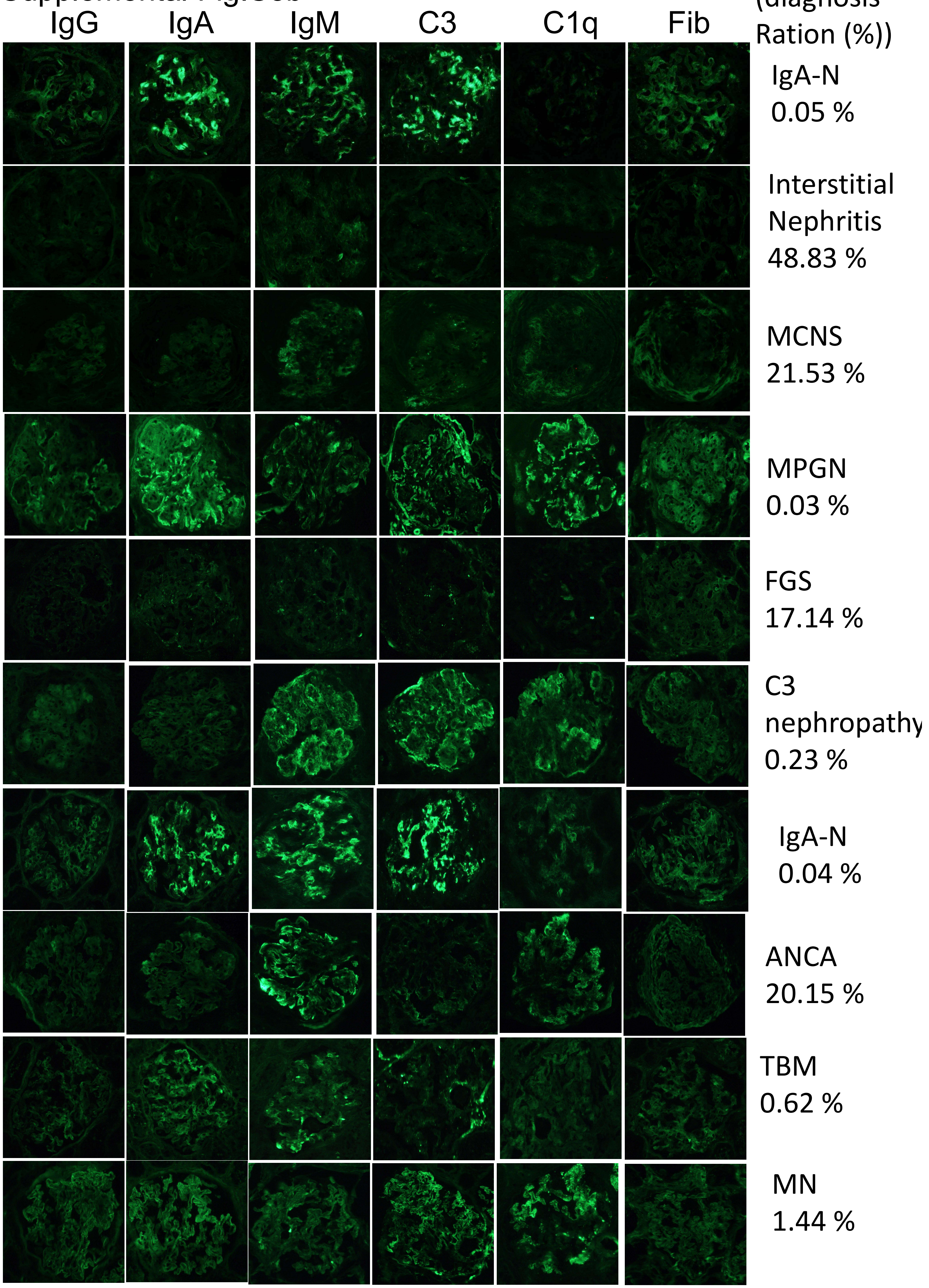

supplementary Figure. S8b
representative false negative (FN) DN diagnosis ration for non DN patients’ images

Supplemental Fig.S9 FP

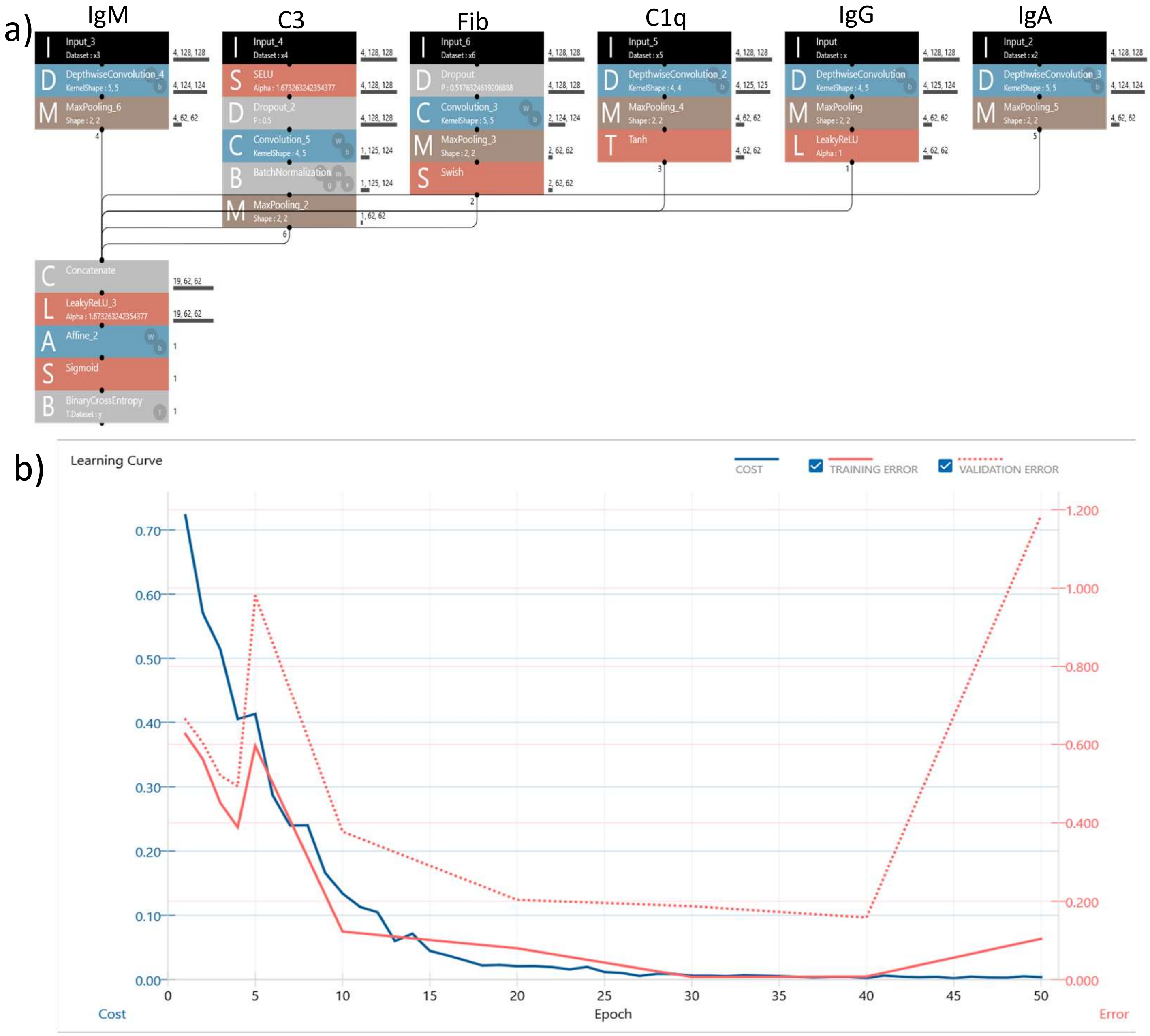

c)

	y'=0	y'=1	Recall
y=0	6	0	1
y=1	3	7	0.7
Precision	0.6666	1	
F-Measures	0.7999	0.8235	

Accuracy	0.8125
Avg.Precision	0.8333
Avg.Recall	0.85
Avg.F-Measures	0.8117

supplementary Figure. S9
representative false positive diagnosis (FP) CNN data
a) schema of CNN, b) learning curve, c) evaluation data
(y=0: DN, y=1: non DN, y`=0: estimated DN, y`=1: estimated non DN)

Supplemental Fig.S10a

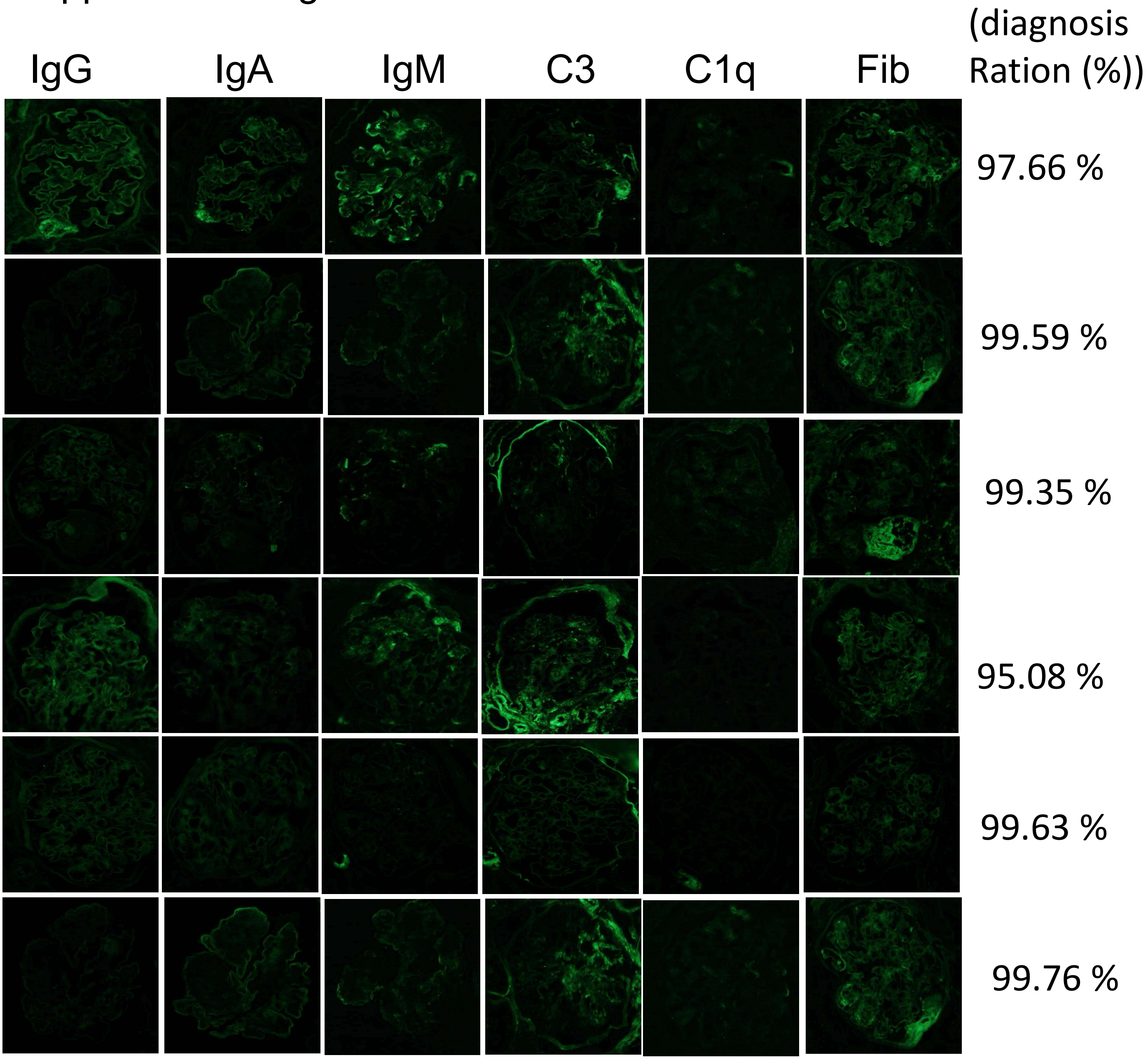

supplementary Figure. S10a
representative false positive (FP) DN diagnosis ration for DN patients' images

Supplemental Fig.S10b

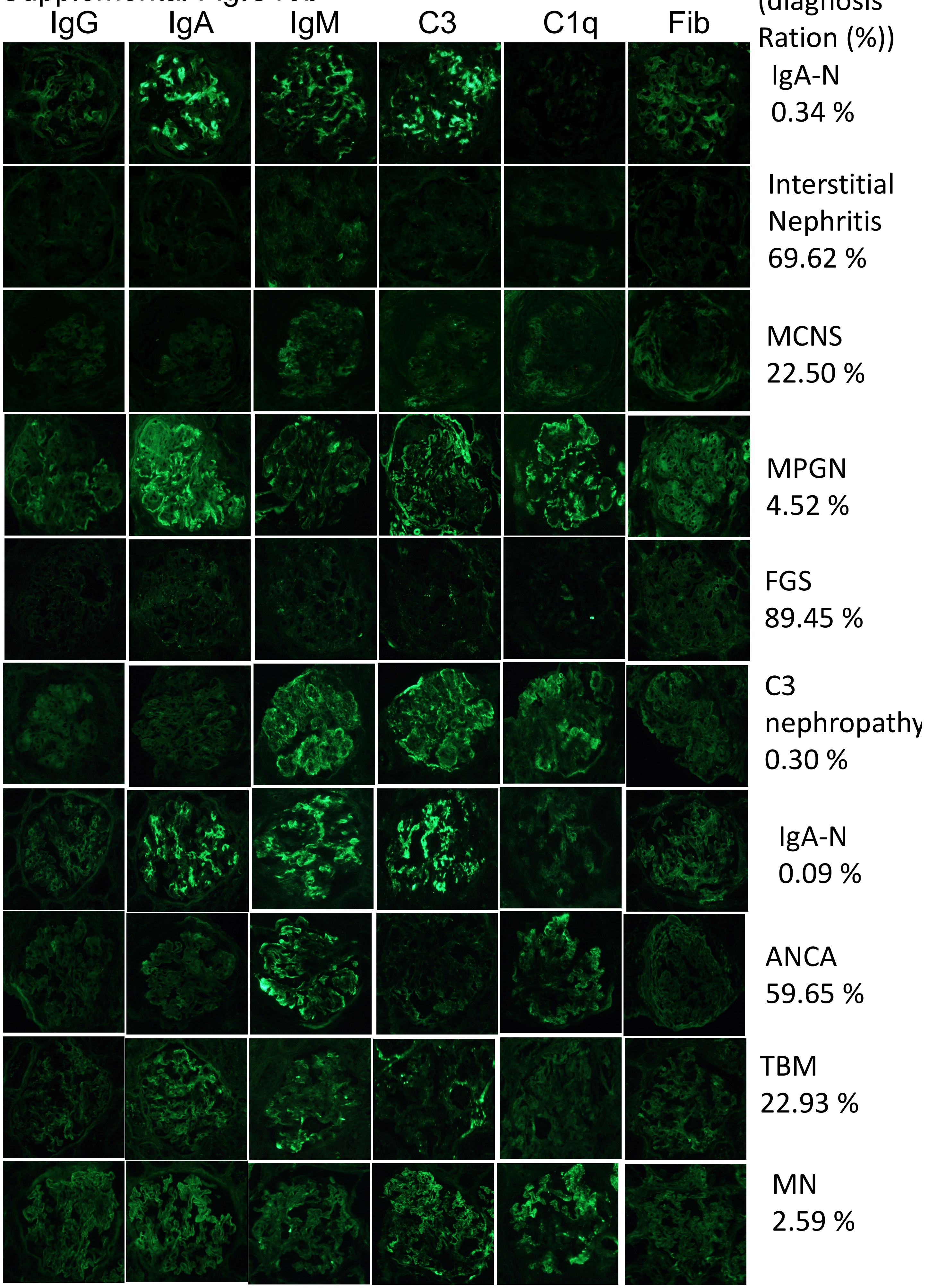

supplementary Figure. S10b
representative false positive (FP) DN diagnosis ration for non DN patients' images

Supplemental Fig.S11 AV

a)

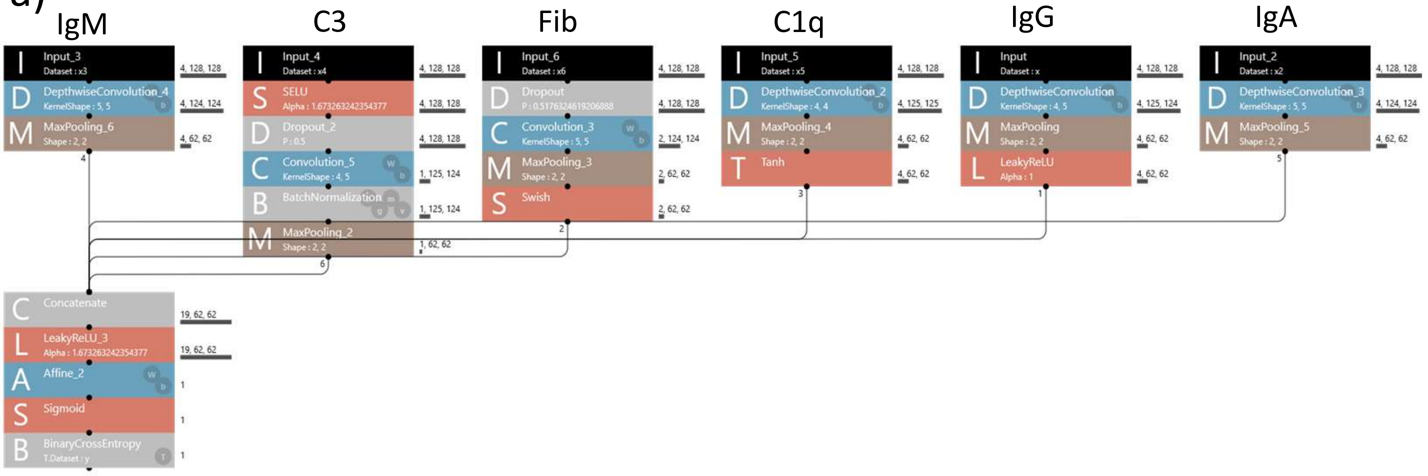

b)

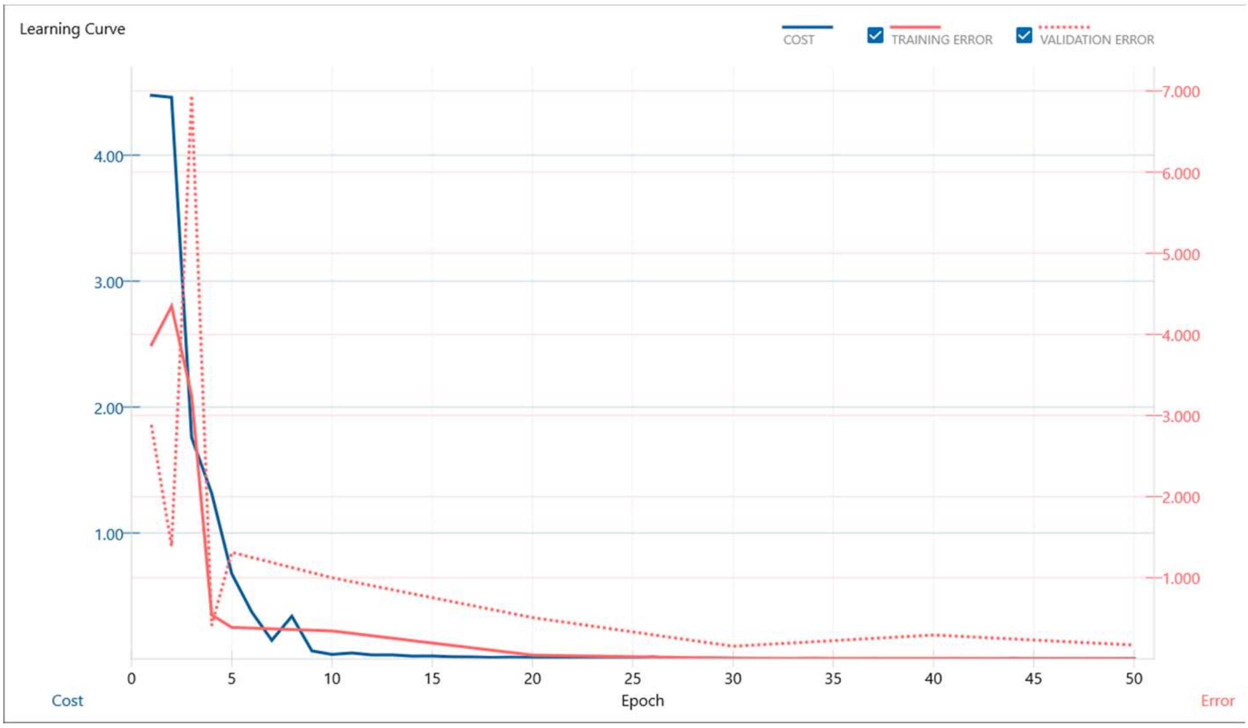

c)

	y'=0	y'=1	Recall
y=0	4	2	0.6666
y=1	1	9	0.9
Precision	0.8	0.8181	
F-Measures	0.7272	0.857	

Accuracy	0.8125
Avg.Precision	0.809
Avg.Recall	0.7833
Avg.F-Measures	0.7921

supplementary Figure. S11
representative average diagnosis (AV) CNN data
a) schema of CNN, b) learning curve, c) evaluation data
(y=0: DN, y=1: non DN, y`=0: estimated DN, y`=1: estimated non DN)

Supplemental Fig.S12a

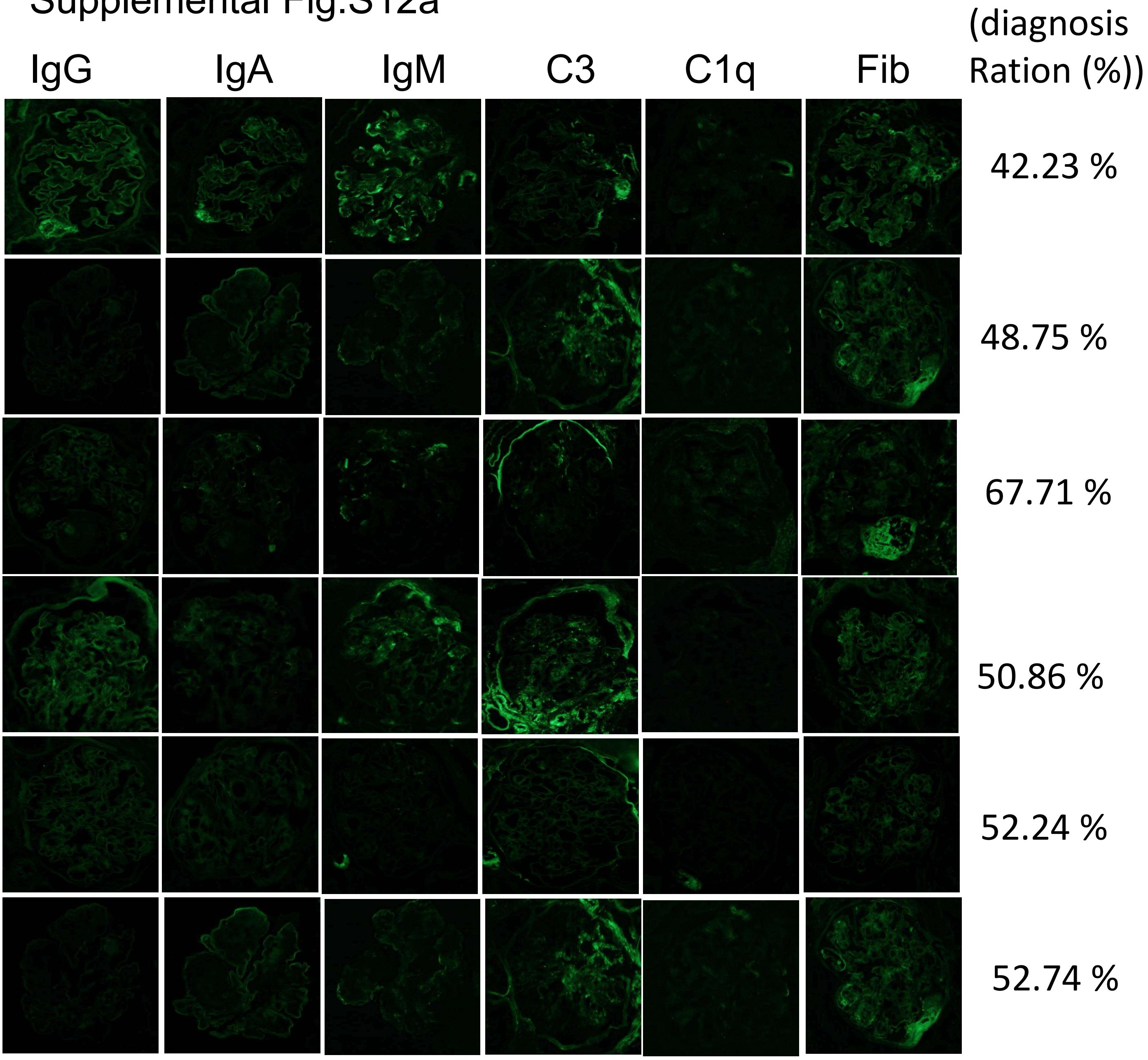

supplementary Figure. S12a
representative average (AV) DN diagnosis ration for DN patients' images

Supplemental Fig.S12b

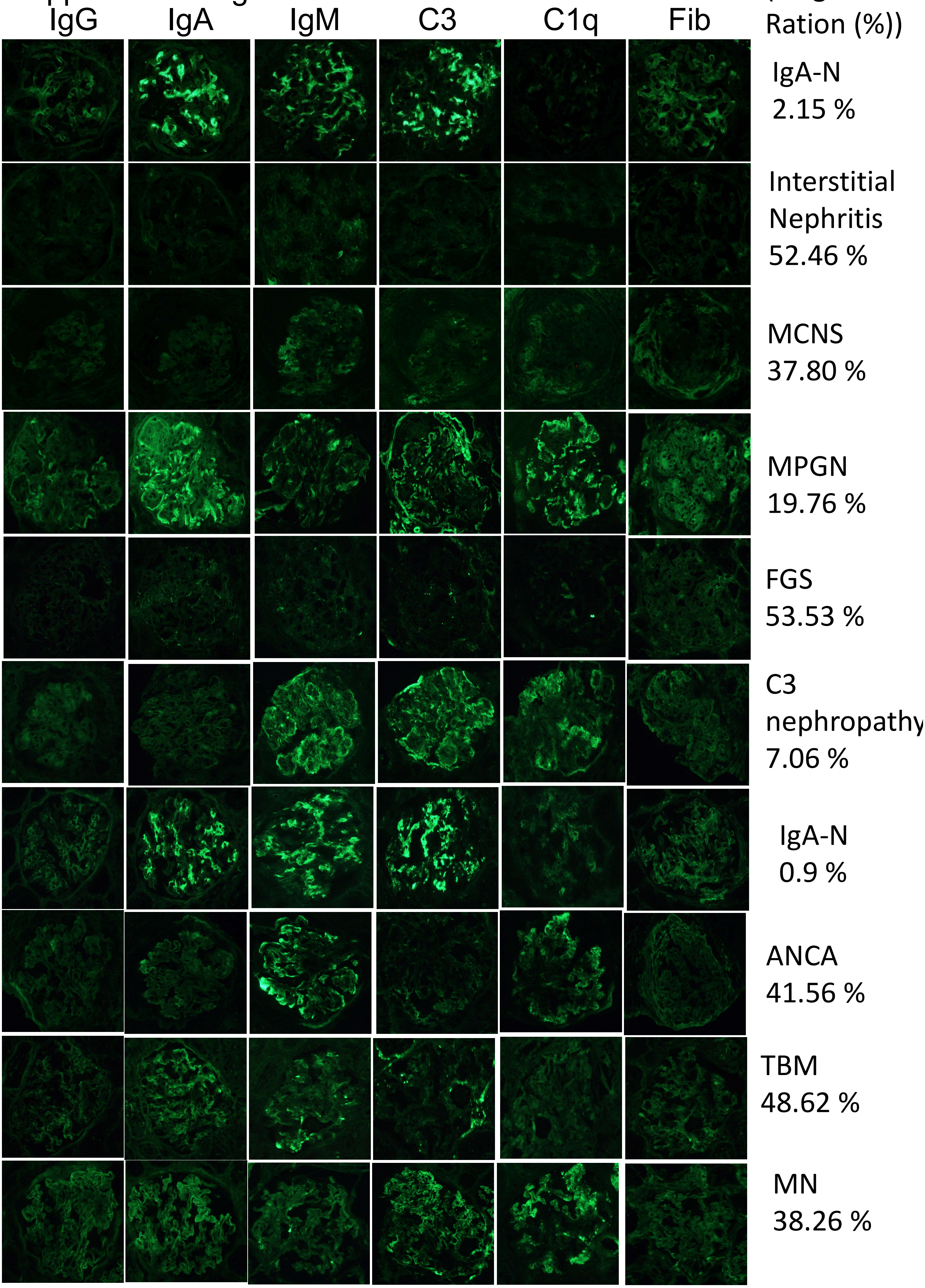

supplementary Figure. S12b
representative average (AV) DN diagnosis ration for non DN patients’ images

Supplemental Fig.S13 CP1

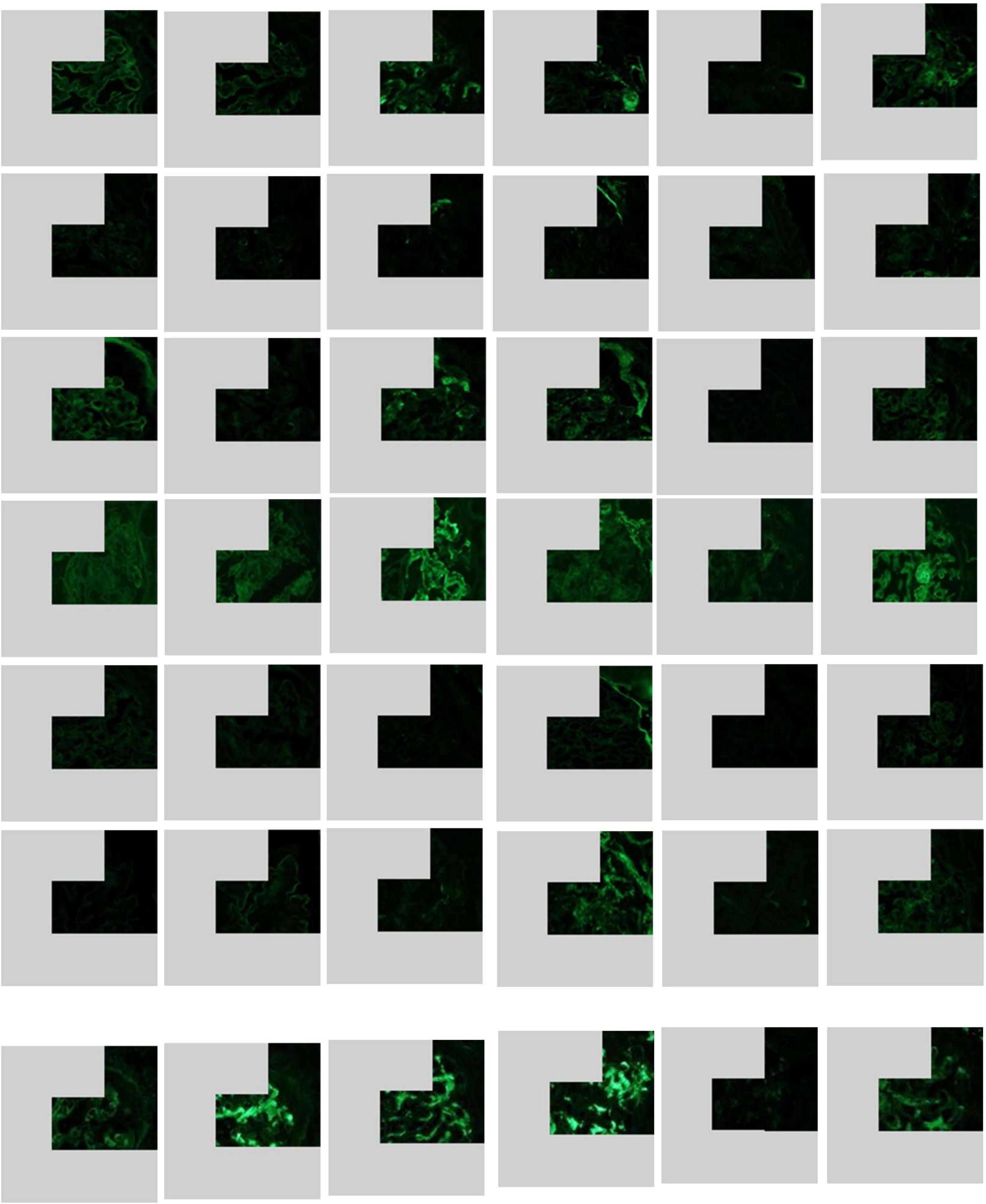

supplementary Figure. S13
Lime analysis for patient #1 images with completely diagnosis program #1

Supplemental Fig.S14

CP6

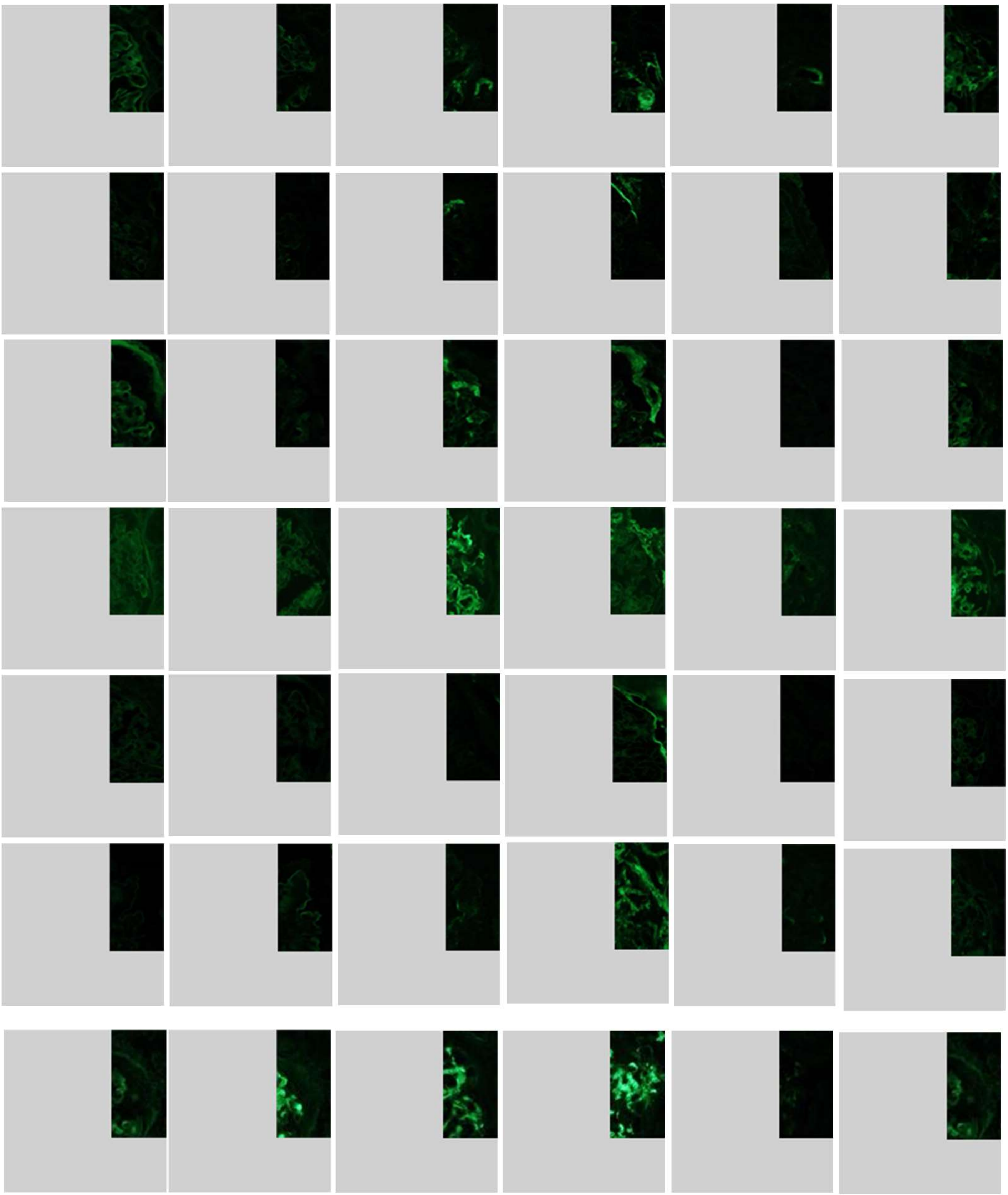

supplementary Figure. S14
Lime analysis for patient #1 images with another completely diagnosis program #6
